# Integration of GWAS and transcriptome approaches for the identification of nitrogen-, phosphorus-, and potassium-responsive genes in tomato

**DOI:** 10.1093/hr/uhaf112

**Published:** 2025-04-24

**Authors:** Mannan Zhang, Huaiqian Tang, Qin Xu, Zhihao Xiao, Chengxuan Zhou, Yuxiao Qian, Ruyue Gong, Huating Zhao, Jiaying Wang, Zijing Xing, Taotao Wang, Bo Ouyang, Yuyang Zhang, Junhong Zhang, Zhibiao Ye, Jie Ye

**Affiliations:** National Key Laboratory for Germplasm Innovation & Utilization of Horticultural Crops, College of Horticulture and Forestry Sciences, Huazhong Agricultural University, Wuhan 430070, China; National Key Laboratory for Germplasm Innovation & Utilization of Horticultural Crops, College of Horticulture and Forestry Sciences, Huazhong Agricultural University, Wuhan 430070, China; National Key Laboratory for Germplasm Innovation & Utilization of Horticultural Crops, College of Horticulture and Forestry Sciences, Huazhong Agricultural University, Wuhan 430070, China; National Key Laboratory for Germplasm Innovation & Utilization of Horticultural Crops, College of Horticulture and Forestry Sciences, Huazhong Agricultural University, Wuhan 430070, China; National Key Laboratory for Germplasm Innovation & Utilization of Horticultural Crops, College of Horticulture and Forestry Sciences, Huazhong Agricultural University, Wuhan 430070, China; National Key Laboratory for Germplasm Innovation & Utilization of Horticultural Crops, College of Horticulture and Forestry Sciences, Huazhong Agricultural University, Wuhan 430070, China; National Key Laboratory for Germplasm Innovation & Utilization of Horticultural Crops, College of Horticulture and Forestry Sciences, Huazhong Agricultural University, Wuhan 430070, China; National Key Laboratory for Germplasm Innovation & Utilization of Horticultural Crops, College of Horticulture and Forestry Sciences, Huazhong Agricultural University, Wuhan 430070, China; National Key Laboratory for Germplasm Innovation & Utilization of Horticultural Crops, College of Horticulture and Forestry Sciences, Huazhong Agricultural University, Wuhan 430070, China; National Key Laboratory for Germplasm Innovation & Utilization of Horticultural Crops, College of Horticulture and Forestry Sciences, Huazhong Agricultural University, Wuhan 430070, China; National Key Laboratory for Germplasm Innovation & Utilization of Horticultural Crops, College of Horticulture and Forestry Sciences, Huazhong Agricultural University, Wuhan 430070, China; National Key Laboratory for Germplasm Innovation & Utilization of Horticultural Crops, College of Horticulture and Forestry Sciences, Huazhong Agricultural University, Wuhan 430070, China; National Key Laboratory for Germplasm Innovation & Utilization of Horticultural Crops, College of Horticulture and Forestry Sciences, Huazhong Agricultural University, Wuhan 430070, China; Hubei Hongshan Laboratory, Wuhan 430070, China; National Key Laboratory for Germplasm Innovation & Utilization of Horticultural Crops, College of Horticulture and Forestry Sciences, Huazhong Agricultural University, Wuhan 430070, China; National Key Laboratory for Germplasm Innovation & Utilization of Horticultural Crops, College of Horticulture and Forestry Sciences, Huazhong Agricultural University, Wuhan 430070, China; Hubei Hongshan Laboratory, Wuhan 430070, China; National Key Laboratory for Germplasm Innovation & Utilization of Horticultural Crops, College of Horticulture and Forestry Sciences, Huazhong Agricultural University, Wuhan 430070, China

## Abstract

Plant growth is inseparable from the presence of mineral nutrients such as nitrogen (N), phosphorus (P), and potassium (K), but the mechanism by which horticultural plants such as tomatoes respond to mineral elements is poorly understood. Here, we collected 28 phenotypic datasets, including 5 agronomic traits and 4 pigment accumulation traits, under full nutrition and nitrogen/phosphorus/potassium-deficiency conditions, most of which showed abundant variation. Phenotyping analysis suggested that the yellowing of leaves under low-nitrogen treatment was caused by an increase in the carotenoid content and a decrease in the chlorophyll *b* content. A genome-wide association study identified a total of 138 suggestive loci (including 23 significant loci) corresponding to 116 loci, including many reported and new candidate genes related to mineral element response and absorption. Transcriptome analysis of tomato seedlings under full nutrient and N/P/K-deficiency conditions revealed 1108 and 1507 common differentially expressed genes in above-ground and below-ground tissues, respectively, with 103 overlapping genes. Gene Ontology term enrichment analysis revealed that tomato plants resist low nutrient stress by increasing photosynthesis in the above-ground parts and ion transport capacity in the below-ground parts. Through the combined analysis of GWAS and RNA-Seq, we identified 28 mineral element response genes with high confidence, corresponding to 17 loci, which may be closely related to the response and utilization of N, P, and K in tomato. Two candidate genes, auxin-repressed protein (*Solyc02g077880*), which responds to carotenoid and chlorophyll *b* accumulation, and guanine nucleotide exchange factor-like protein (*Solyc04g005560*), which responds to low-phosphorus conditions, were further validated via haplotype analysis. This study provides new insights into the nitrogen, phosphorus, and potassium response mechanisms of tomato and offers valuable genetic resources for future improvements in tomato breeding.

## Introduction

Plants require at least 14 essential mineral nutrients and several beneficial elements to maintain their growth, development, and resistance to biotic and abiotic stresses, with nitrogen (N), phosphorus (P), and potassium (K) being the most critical macronutrients [1]. These elements play vital roles in various physiological processes within plant cells, including photosynthesis, enzyme activation, protein and nucleotide synthesis, osmoregulation, cell expansion, and ion homeostasis [2]. For example, low N stress limits chlorophyll synthesis by decreasing the supply of glutamine and inhibiting the activity of multiple enzymes in the chlorophyll synthesis pathway, thereby leading to leaf yellowing [3]. Moreover, the deficiency of N, P, and K in poor soils not only limits plant growth but also adversely affects yield and quality. To meet the demand of high yields in modern agriculture, chemical fertilizers containing N, P, and K are frequently applied in large quantities, resulting in low nutrient utilization efficiency and severe damage to the ecological environment [4]. In the case of nitrogen fertilizers, plants absorb only 25% to 50% of the applied nitrogen [5]. In addition, overfertilization can also lead to other harmful effects. For example, in tomatoes, N regimes can alter the metabolome of tomato leaves and their interactions with pests. High nitrogen fertilization has been associated with increased attraction of whiteflies (*Bemisia tabaci*) due to changes in the emission of volatile compounds [6]. Moreover, excessive fertilization does not significantly increase tomato yield; instead, it can decrease fruit quality by reducing sugar content and increasing acidity [7]. Therefore, reducing the application of macronutrient fertilizers while maintaining crop yield and quality is an urgent challenge.

Considerable progress has been made in understanding the response mechanisms of the model plants *Arabidopsis thaliana* and crops under N-, P-, and K-deficiency conditions. In fact, plants have evolved many adaptive response mechanisms in response to low N, P, and K stress. When plants receive low N, P, and K signals, many adaptive changes occur to maintain growth and development, and N, P, and K use efficiency can be improved by changing the root structure and element absorption and transport system, which involves the expression and regulation of many stress response genes [8–10].

Sixteen transcription factors (TFs) that play a role in N metabolism have been identified in *Arabidopsis*, seven of which are nitrogen-dependent factors for root development [11]. Through yeast one-hybrid and metabolic analyses, a transcriptional regulatory network associated with N metabolism containing 21 TFs that respond effectively to environmental N changes by regulating the structure of plant roots and stems, was established [12]. In addition, a number of genes are thought to improve N use efficiency and yield in crops, including *ARE1*, *DEP1*, *GRF40-MYB61*, *NAC42-OsNPF6.1*, *OsNR2*, *OsTCP19,* and *OsLBD*, and the functional alleles of these genes achieve increased and stable yields under low N growth conditions [13–18]. P has lower mobility and weaker diffusion ability, resulting in a limited distribution in soils. Under low-P stress, plants produce more root hairs to better absorb P from the soil, and auxin and cytokinin play regulatory roles in this process [19]. P transporters (PHTs) have a strong affinity for phosphorus and play important roles in regulating the absorption and transport of phosphorus in plants, such as *OsPHT1;8*, which is involved in auxin and the phosphate starvation response and regulates phosphorus distribution between the embryo and endosperm in seeds, and *OsPHT1;7*, which plays a role in phosphorus accumulation and redistribution in anthers [20–22]. K is present in plants as soluble ions (K^+^) and plays a vital role in many physiological processes, including osmoregulation, protein biosynthesis, and the transport of assimilated products [23]. A number of K^+^ transporters have been identified in crops, such as *OsHAK1*, *OsAKT1*, *OsHAK5,* and *OsHAK21* in rice and *ZmHAK5* and *ZmHAK1* in maize [24–26]. Many K^+^ transporters are involved in multiple physiological processes, such as K^+^ absorption, transport, and distribution, and are regulated and modified in the process of transcription and translation [27]. Low-K stress affects the growth of root hairs and primary roots through its involvement in ethylene signal transduction [28], and numerous genes or quantitative trait locus (QTLs) associated with low-K resistance have been identified in rice [29, 30], wheat [31], and *Arabidopsis* [32], with root length used as an indicator.

Compared with those of crops, the overall identification of vegetable responses to macronutrients (N, P, and K) remains significantly limited. Tomato (*Solanum lycopersicum*) is one of the most important vegetable crops worldwide and has high nutritional and commercial value. Proper application of fertilizer is beneficial for the growth and yield of tomatoes. Genome-wide association studies (GWASs) have proven to be an effective method for investigating the genetic architecture of complex agronomic traits and for identifying corresponding loci or candidate genes in tomatoes [33]. Additionally, combining transcriptomic analysis with GWAS is a powerful method for detecting genetic factors and transcriptional regulatory mechanisms of different biological processes [4, 34].

Here, we investigated the response of tomatoes to low N, P, and K at the seedling stage by measuring five development-related traits and four pigment contents in a natural population composed of 427 diverse tomato accessions collected from around the world. A total of 138 genomic loci were identified by genome-wide association study analysis of these traits, of which six were known genomic loci for N-, P-, and K-deficiency responses and five were reported genomic loci for plant height, for example, *qtph5.1*, and *qth11.1* [35–38]. Additionally, analysis of DEGs between the above-ground and below-ground parts under normal and low N, P, and K conditions revealed more precise candidate genes, and two genes were further verified by haplotype analysis. Overall, our findings provide new insights into how tomato plants respond to N, P, and K stress and provide valuable resources for future tomato breeding and improvement.

## Results

### Phenotypic variation in natural tomato populations under full nutrient and low N, P, and K stress

To systematically dissect the genetic basis of the N-, P-, and K-deficiency response of tomato, a natural population of 427 diverse tomato accessions was evaluated for various growth traits under full nutritional, N-, P-, and K-deficiency conditions at the seedling stage [33]. This genotyped population included 31 *Solanum pimpinellifolium* accessions (SPIM = 31), 172 *S. lycopersicum* var. *cerasiforme* accessions (SLC = 172) and 224 *S. lycopersicum* accessions (SLL = 224) (Supplementary Table S1). N-, P-, and K-deficiency treatments were performed on 2-week-old seedlings for 2 weeks. A total of 28 phenotypic datasets were used, including plant height (PH), above-ground tissues fresh weight (AFW), above-ground tissues dry weight (ADW), below-ground tissues fresh weight (BFW), and below-ground tissues dry weight (BDW) under full nutrition (CK_PH, CK_AFW, CK_ADW, CK_BFW, CK_BDW), low N stress conditions (LN_PH, LN_AFW, LN_ADW, LN_BFW, LN_BDW), low P stress conditions (LP_PH, LP_AFW, LP_ADW, LP_BFW, LP_BDW), low K stress conditions (LK_PH, LK_AFW, LK_ADW, LK_BFW, LK_BDW), and the contents of carotenoids (*CAR*), chlorophyll *a* (*Chla*), chlorophyll *b* (*Chlb*), and total chlorophyll (*Chl*(*a + b*)) in young leaves under full nutrition (CK_*CAR*, CK_*Chla*, CK_*Chlb*, CK_*Chl*(*a + b*)) and low N stress conditions (LN_*CAR*, LN_*Chla*, LN_*Chlb*, LN_*Chl*(*a + b*)) (Supplementary Table S2-S3). These datasets can be classified into three categories: 4 length-related traits, 16 weight-related traits, and 8 pigment content traits. For the majority of the traits, a large range of variation was detected, with coefficients of variation (CVs) ranging from 5.00% for LN_*CAR* to 114.27% for LP_ADW. Most traits followed a normal distribution, whereas traits such as CK_AFW, CK_*Chla*, CK_BDW, LN_*CAR*, LP_ADW, LP_AFW, LP_ADW, LP_BDW, LK_ADW, and LK_BDW exhibited skewed distributions (Supplementary Fig. S1).

**Figure 1:**
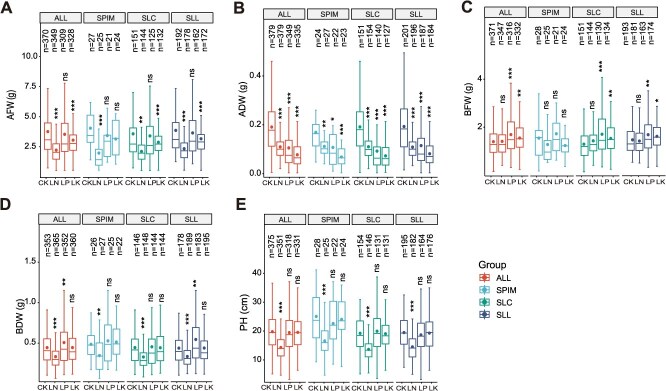
Phenotypic variation of natural tomato populations under the CK, LN, LP, and LK conditions. (A) Above-ground tissue fresh weight. (B) Above-ground tissue dry weight. (C) Below-ground tissue fresh weight. (D) Below-ground tissue dry weight. (E) Plant height. CK: full nutrient, LN: low nitrogen stress, LP: low phosphorus stress, LK: low potassium stress. Asterisks indicate significant differences according to the *t* test: * *P* < 0.05, ** *P* < 0.01, *** *P* < 0.001. ALL: tomato natural populations, SPIM, *S. pimpinellifolium.* SLC, *S. lycopersicum var. cerasiforme*. SLL, *Solanum lycopersicum var. lycopersicum*.

To research the changes in the response to N, P, and K deficiency during tomato evolution, the phenotypic variation among three subgroups of tomatoes (SPIM, SLC, and SLL) was analyzed. We observed that after LN, LP, and LK conditions, the AFW and ADW of the three subgroups significantly decreased, whereas the BFW and BDW significantly increased; the *Chl*(*a + b*), *Chlb*, and *CAR* contents were significantly decreased after the LN treatment (Supplementary Table S3). However, different tomato subgroups differed in their response to LN, LP, and LK stress conditions. For example, after LN conditions, the AFW and BFW of the SPIM subgroup did not differ from those of CK, while the AFW and BFW of the SLL subgroup and SLC subgroup significantly decreased. The BDW of the SPIM subgroup and SLC subgroup did not differ from that of CK, but the BDW of the SLC subgroup significantly increased. In addition, after LP conditions, the BFW of the SPIM subgroup did not differ from that of the CK subgroup, whereas the BFW of the SLL subgroup and SLC subgroup significantly increased (Fig. 1 and Supplementary Fig. S2). These results suggested that the response to LN, LP, and LK was modified during tomato evolution.

Correlation analysis of the 28 traits revealed that numerous traits exhibited significant correlations, with several pairs demonstrating very high correlation coefficients (*r*). Notably, the correlation between LP_AFW and LP_PH was high (*r* = 0.74), as was the correlation between LN_AFW and LN_BFW (*r* = 0.75). Furthermore, the correlation between CK_*Chlb* and CK_*Chl*(*a + b*) was exceptionally strong (*r* = 0.90) (Supplementary Fig. S3). Interestingly, the LN, LP, and LK conditions significantly influenced the correlations among traits. For both length-related and weight-related traits, stress conditions led to an overall increase in the correlation between PH and SFW, as well as between AFW and BFW. For example, the correlation coefficients increased from 0.20 for CK_PH and CK_BFW to 0.50 for LN_PH and LN_BFW, from 0.29 for CK_AFW and CK_BFW to 0.75 for LN_AFW and LN_BFW, and to 0.49 for LP_AFW and LP_BFW, and to 0.3 for LK_AFW and LK_BFW. These findings suggest that when plants experience stress conditions due to LN, LP, and LK, their response strengthens the relationship between the above-ground and below-ground tissues to maintain growth and development. LN stress causes leaf yellowing because nitrogen is the main component of chlorophyll, and approximately 80% of the nitrogen in leaves is allocated to chloroplasts [39]. For pigment-related traits, under LN stress, the correlations among *CAR*, *Chla*, and *Chlb* showed the opposite relationship to that of the full nutrient mixture (*r* = −0.14 between CK_*CAR* and CK_*Chla* and *r* = 0.33 between LN_*CAR* and LN_*Chla*; *r* = −0.42 between CK_*CAR* and CK_*Chlb* and *r* = 0.60 between LN_*CAR* and LN_*Chlb*). These findings suggest that LN stress affects the synthesis and accumulation of *Chla*, *Chlb*, and *CAR* in tomato leaves.

**Figure 2:**
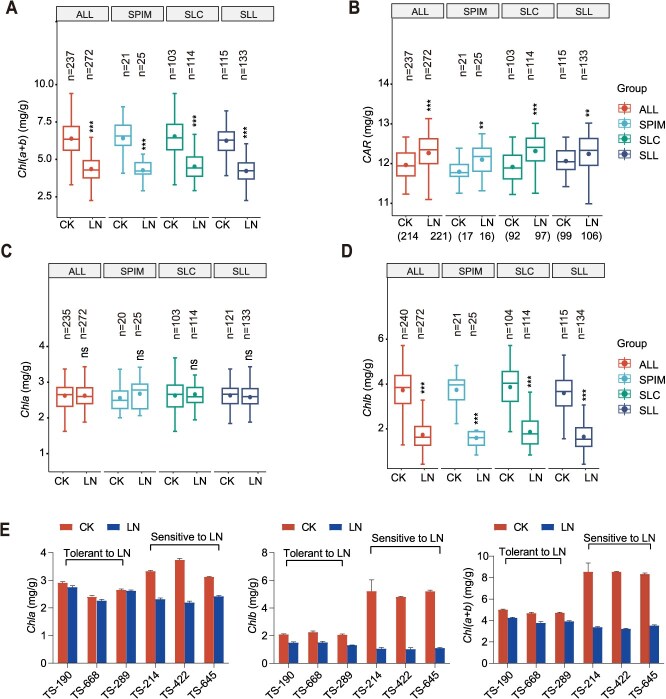
Variation in the pigment contents of natural tomato populations under CK and LN conditions. (A) Chlorophyll (*a + b*) content. (B) Carotenoid content. (C) Chlorophyll *a* content. (D) Chlorophyll *b* content. (E) Pigment contents of LN-tolerant materials and LN-sensitive materials under CK and LN conditions. CK: full nutrient, LN: low nitrogen stress. Asterisks indicate significant differences according to the t test: * *P* < 0.05, ** *P*< 0.01, *** *P* < 0.001. ALL, tomato natural populations; SPIM, *S. pimpinellifolium*. SLC, *S. lycopersicum var. cerasiforme*. SLL, *Solanum lycopersicum var. lycopersicum*.

Leaf yellowing caused by low N is a common phenomenon in nature. In tomato, we found that leaf yellowing caused by low nitrogen was caused by reduced *Chlb* content and increased *CAR* content, whereas the *Chla* content did not change. Under LN conditions, the contents of *Chl*(*a + b*) and *Chlb* in the leaves of 3 tomato subgroups (SLL, SLC, and SPIM) and the whole population significantly decreased, and the contents of *CAR* significantly increased (Fig. 2A–D). The relative percentage (RVP = (CK−LN)/CK) of *Chl*(*a + b*) and *Chlb* could reflect the tolerance of tomato to LN; the higher the relative change rate, the greater the change in LN relative to CK, that is, the LN-sensitive material. The lower the relative change rate, the smaller the change in LN to CK, that is, the material with LN tolerance. Clustering analysis of the RVP of the *Chl*(*a + b*) and *Chlb* contents under the LN and CK conditions revealed that the LN-sensitive and LN-tolerant materials clustered (Supplementary Fig. S4). Three extremely LN-tolerant materials (TS-645, TS-422, and TS-214) and three extremely LN-sensitive materials (TS-190, TS-688, and TS-289) were selected for LN conditions. Chlorophyll content analysis revealed that under LN stress, the content of *Chla* in extremely LN-tolerant materials remained unchanged, whereas the contents of *Chlb* and *Chl*(*a + b*) decreased. In contrast, extremely LN-sensitive materials exhibited reductions in the contents of *Chla*, *Chlb*, and *Chl*(*a + b*) under LN conditions, with the most pronounced decrease observed in the *Chlb* content (Fig. 2E). These findings suggest that the decline in chlorophyll content under LN stress is due primarily to the reduction in *Chlb* content.

Overall, compared with full nutrition, LN treatment led to significant alterations in PH, AFW, ADW, BDW, *CAR*, *Chlb*, and *Chl*(*a + b*). Similarly, LP treatment resulted in notable changes in BFW, ADW, and BDW. LK conditions, on the other hand, caused notable changes in the AFW, BFW, and BDW. Collectively, these results indicate that stress conditions induced by LN, LP, and LK have a significant effect on tomato growth and development.

### Genome-wide association studies of 28 phenotypic datasets

To elucidate the genetic architecture of tomatoes in response to mineral elements (N, P, and K), we conducted a GWAS for 28 phenotypic datasets using a reported genotypic dataset consisting of 322 580 SNPs generated for the 427 accessions [40]. In this study, genome-wide association studies (GWASs) were conducted using the PCA + K model implemented in EMMAX software. The population structure was corrected using two different PCA approaches: PCA5 and PCA10. The significance thresholds were established through Bonferroni correction, with suggestive and significant thresholds set at 3.1 × 10^−6^ and 1.55 × 10^−7^, respectively. Comparative analysis revealed that the PCA10 + K model exhibited greater detection power for significant loci than did the PCA5 + K approach (Supplementary Fig. S5). Consequently, the results obtained from the PCA10 + K model were selected for subsequent analyses. Detailed Manhattan plots and QQ plots for all 28 phenotypic datasets are shown in Supplementary Figs S6–S33, and all significant associations are summarized in Supplementary Table S4.

**Figure 3:**
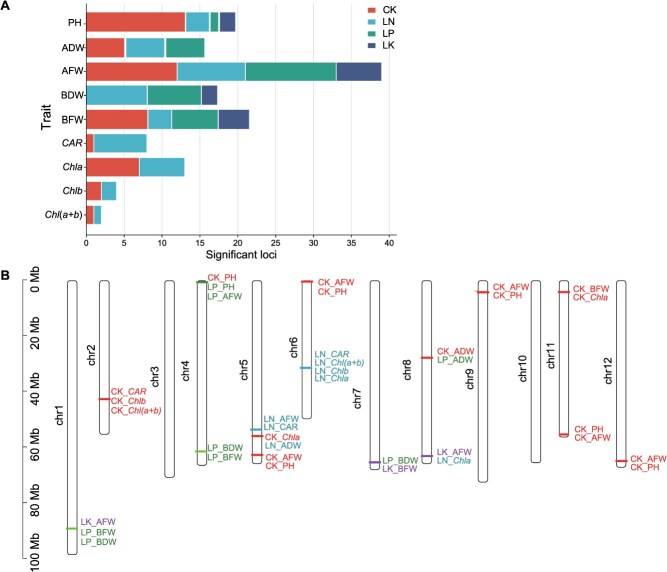
Statistical analysis of significantly associated loci for 9 phenotypes under different conditions. (A) The significantly associated loci were simultaneously detected under CK, LN, LP, and LK conditions. (B) Distribution of 16 common loci that were repeatedly detected within or across locations on 12 chromosomes according to physical distance. The number on the left side of each column represents the physical location (Mb) of each lead SNP. The letters to the right of the column represent the corresponding trait.

In our study, association loci were defined as chromosomal regions where the distance between adjacent lead SNPs was less than 200 kb, and the SNP with the lowest *P*-value at a locus was defined as the lead SNP [41]. According to this definition, a total of 138 suggestive associations (including 23 significant associations) corresponding to 116 loci were identified (Fig. 3A). There were 44, 31, and 14 loci associated with LN, LP, and LK stress, respectively, of which nine loci were simultaneously associated with different deficiency conditions (Fig. 3). Comparison analysis revealed that the number of significant loci for CK_AFW (a total of 19 loci) was greater than that for the other datasets. Additionally, except for BDW and *CAR*, the number of loci detected under full nutrition was greater than that detected under LN, LP, and LK stress across all datasets. Sixteen potential GWAS hotspots associated with at least two traits were identified, which closely matched known QTLs involved in tomato growth and nutrient-deficiency response. For example, the loci overlapping with *qtph5.1* and *qtph11.1* were simultaneously detected in CK_PH and CK_AFW. *qtph5.1* and *qtph11.1* are the main loci regulating tomato PH [37]. Under LN conditions, L58 was simultaneously detected in LN_*CAR*, LN_*Chla*, LN_*Chlb*, and LN_*Chl*(*a + b*), and L109 and L110 were detected in LN_AFW and LN_BDW. L58, L109, and L110 overlapped with the loci detected in previous studies for leaf nitrogen content and petiole nitrate content under LN conditions [35]. These results are consistent with the observation that many of the traits were highly correlated (Supplementary Fig. S3). The percentage of phenotypic variation explained by each locus ranged from 5.70% to 10.58% for the pigment traits, from 0.66% to 10.61% for the weight datasets, and from 6.55% to 9.33% for the length datasets, with mean values of 7.12%, 8.53%, and 7.26%, respectively (Supplementary Table S5). Most traits were determined by multiple moderate-effect loci (Supplementary Table S5).

Candidate genes were detected within a 200-kb upstream and downstream range of the significant lead SNPs (Supplementary Table S4). For example, for L105, which was associated with LN_PH, the *Solyc12g005310* gene (*SlGH3.15*) is located 167 kb downstream of lead_SNP SL2.50chr12_353321. This gene belongs to the glycoside hydrolase 3 family, one of the crucial early auxin-responsive gene families, and plays an important regulatory role in plant growth, development, and stress response [42]. Among the candidate genes in L11, *Solyc01g090270* has a homologous gene in *Arabidopsis*, *AT3G60320* (*NRG2*), which is responsible for regulating nitrate signaling. The overexpression of *AtNRG2* can promote plant growth and improve nitrogen use efficiency [43]. In addition, a total of 77 genes were detected in the upstream and downstream 200 kb range of L30, which was associated with LP_PH, LP_AFW, and CK_PH. Among these candidate genes, *Solyc04g005560* encodes a guanine nucleotide exchange factor-like protein. The homologous gene in *Arabidopsis*, *AT1G13980,* is involved in the accumulation of auxin in lateral roots, which in turn controls the direction of root growth. It also regulates phototropism to affect plant biomass accumulation [44, 45]. The new and reported loci identified here provide valuable resources for nutrient-efficient breeding of tomatoes.

### Transcriptome analysis identification of DEGs under LN, LP, and LK stress

**Figure 4:**
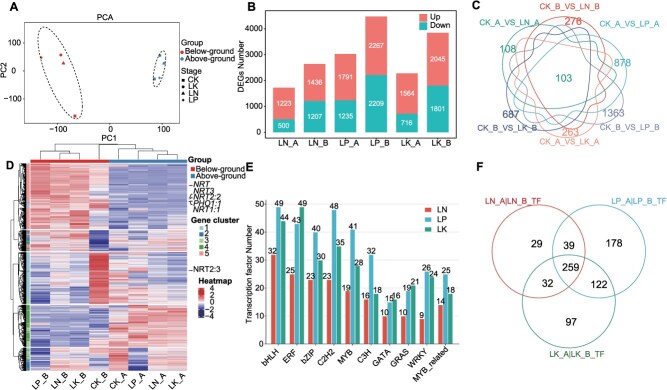
Variability in transcript levels among above-ground tissues and below-ground tissues under CK, LN, LP, and LK conditions. (A) PCA results of the transcriptome data. (B) The number of DEGs in the LN, LP, and LK conditions in the above-ground tissues and below-ground tissues compared with those in the CK conditions. (C) Overlap of DEGs in LN, LP, and LK conditions in above-ground tissues and below-ground tissues compared with CK conditions. (D) Heatmap of gene expression in the above-ground tissues and below-ground tissues under the CK, LN, LP, and LK conditions. NRT, nitrogen transporter; PHO, phosphate transporter. (E) The number of transcription factors (TFs) in LN, LP, and LK conditions in above-ground tissues and below-ground tissues compared with CK conditions. (F) Overlap of TFs in LN, LP, and LK conditions in above-ground tissues and below-ground tissues compared with CK.

RNA-Seq represents an effective approach for performing genome-wide investigations of gene expression and dissecting the gene regulatory networks associated with complex agronomic traits. We performed RNA-Seq of above-ground and below-ground tissues to identify response genes in tomatoes under 24 h of LN, LP, and LK conditions. A total of 49.89 Gb of clean data were generated, with up to 6.23 Gb of clean data per sample. Approximately, 92.47% of the clean reads had a quality score ≥ Q30 (≤0.1% error rate), indicating high read quality. Approximately, 92.44% to 96.39% of the clean reads were mapped to the tomato reference genome using HISAT2 software, with an average mapping rate of 94.82%. PCA grouped the samples into two distinct clusters, aboveground and belowground (Fig. 4A), suggesting that the differences in gene expression levels between different tissues are more pronounced than those under LN, LP, and LK stress. To verify the accuracy of the RNA-Seq data, we selected several previously reported marker genes that respond to LN, LP, and LK for qRT–PCR analysis [46, 47], and the results revealed that these marker genes were significantly upregulated under nutrient-deficiency stress, which was consistent with the RNA-Seq results (Supplementary Fig. S34).

Using Gfold software with a |log2-fold change| > 1 as the threshold [48], DEGs were identified in above-ground and below-ground tomato tissues under LN and LP LK stress compared with those under full nutrition. Under LN stress, 1723 and 2643 DEGs were identified in the above-ground and below-ground tissues, respectively. For LP stress, 3026 DEGs were found in the above-ground tissues and 4476 DEGs were found in the below-ground tissues. LK stress resulted in 2280 DEGs in the above-ground tissues and 3846 DEGs in the below-ground tissues. The number of DEGs in the below-ground tissues under LN, LP, and LK stress conditions was significantly greater than that in the above-ground tissues, with the below-ground tissues containing 1.52, 1.47, and 1.69 times more DEGs, respectively (Fig. 4B, Supplementary Table S6). This increase is likely due to the direct role of below-ground tissues in the absorption and utilization of major elements. The number of DEGs shared in above-ground and below-ground tissues under LN, LP, and LK stress was 1108 and 1507, respectively, accounting for 64.31% and 57.02%, 36.62% and 33.67%, 48.60% and 39.18% of the DEGs in above-ground and below-ground tissues under LN, LP, and LK stress, respectively, indicating that the genetic responses of tomatoes to LN, LP, and LK stresses were similar at the transcriptional level (Supplementary Table S6). Moreover, there were 103 common DEGs in both above-ground and below-ground tissues under all three stresses, including 56 upregulated DEGs in both tissues and 15 downregulated DEGs in both tissues, and the remaining 29 DEGs showed opposite changes between the two tissues (Fig. 4C, Supplementary Fig. S35, Supplementary Table S7). *Solyc02g085630* is annotated as a homeobox-leucine zipper-like protein that has a highly similar sequence to *AtHB40* (*AT4G36740*), and *AtHB40* has been reported to regulate gibberellin (GA) homeostasis [49]. In our study, the expression of *Solyc02g085630* in above-ground tissues and below-ground tissues decreased under LN, LP, and LK stress conditions.

To further investigate the gene expression patterns of tomatoes under CK, LN, LP and LK stress conditions, we analyzed the entire set of DEGs and identified five distinct expression modules (Fig. 4D, Supplementary Fig. S36, Supplementary Table S8):

- Cluster 1: Genes highly expressed under CK conditions
- Cluster 2: Genes downregulated under LN, LP, and CK conditions
- Cluster 3: Genes highly expressed under CK conditions
- Cluster 4: Genes expressed at higher levels in the above-ground tissues than in the below-ground tissues
- Cluster 5: Genes expressed at lower levels in the above-ground tissues than in the below-ground tissues

Some of these DEGs are known or predicted to be involved in nitrogen, phosphorus, and potassium utilization, such as *Solyc08g078950* (*SlNRT1:1*), *Solyc06g010250* (*SlNRT2:2*)*, Solyc06g074990* (*SlNRT2:3*)*, Solyc07g032490* (*SlNRT3*)*, Solyc11g069740* (*SlNRT*)*,* and *Solyc09g090360* (*SlPHO1:1*) [50–52]*.* These genes are highly expressed in below-ground tissues, indicating the reliability of the transcriptome data.

Gene ontology (GO) term enrichment analysis revealed that DEGs in the above-ground tissues under LN, LP, and LK stress conditions were significantly enriched in several functional categories, including photosynthesis, response to light intensity, light and electron transport chain, pigment biosynthesis, response to blue light, circadian rhythm, precursor metabolism, and energy production (Supplementary Fig. S37). These findings indicate that, under initial LN, LP, and LK stress conditions, photosynthesis in above-ground tissues is increased to facilitate energy conversion and metabolite synthesis, thereby maintaining normal growth and development. For example, *Solyc02g070940* and *Solyc06g066640* were annotated as chlorophyll a-b binding protein and photosystem I reaction center subunits, which were upregulated in LN_A (from 542.87 and 57.74 to 5611.49 and 214.50), LP_A (from 542.87 and 57.74 to 7809.98 and 312.09), and LK_A (from 542.87 and 57.74 to 4859.89 and 182.82). In contrast, DEGs in the below-ground tissues under LN, LP, and LK stress conditions were significantly enriched in GO terms related to the metal ion response, ion transport, inorganic anion transport, positive regulation of ion transporters, and secondary metabolite biosynthesis processes. For example, the expression of genes encoding nitrate transporters and sulfate transporters (*Solyc00g090860* and *Solyc12g056920*) increased in LN_B (from 6.95 and 25.37 to 57.57 and 175.19), LP_B (from 6.95 and 25.37 to 123.61 and 210.78), and LK_B (from 6.95 and 25.37 to 97.98 and 149.43). These findings suggest that during the early stages of LN, LP, and LK stress, the below-ground tissues focus on processes related to ion transport to improve the efficiency of mineral element uptake by below-ground tissues, thereby ensuring normal metabolic functions (Supplementary Figs S37–S38).

TFs play a critical role in controlling plant growth and development through the regulation of gene expression. In this study, we performed TF prediction for DEGs under LN, LP and LK stress conditions. A total of 359, 598, and 510 differentially expressed TFs were identified under LN, LP, and LK stress conditions, respectively (Fig. 4E, Supplementary Table S9), corresponding to 50 different TF families. Among these, 259 predicted TFs were commonly expressed under all three stress conditions (Fig. 4F). The TF predictions for DEGs provide preliminary insights into key TF families in tomato, such as BHLH, GRAS, BZIP, and ERF, which play crucial roles in early responses to LN, LP and LK stress conditions. For example, *Solyc12g005340* is a GRAS family TF, and its homolog gene in *Arabidopsis* is *AtPAT1* (*AT5G48150*). In this study, we found that the expression of *Solyc12g005340* increased in the above-ground and below-ground tissues under LN, LP, and LK stress conditions. *AtPAT1* has been reported to be involved in the plant response to UV-B and far-red light mediated by phytochrome A (PHYA) [53, 54].

**Figure 5:**
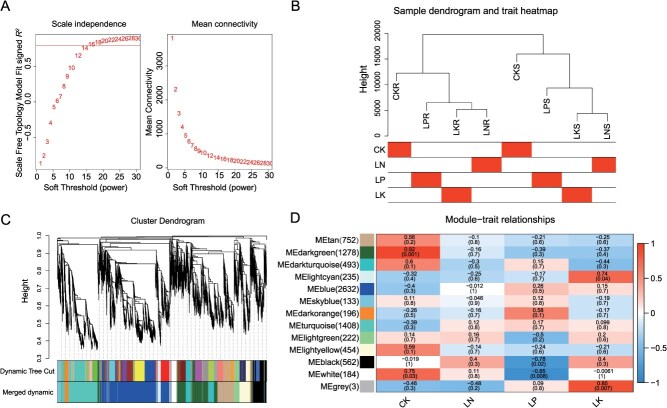
Gene expression clustering and coexpression analysis of the transcriptome. (A) The expression of network topology characteristics under different soft thresholds. (B) Sample dendrogram and heatmap of different stress environments. (C) Clustering tree of the coexpression module. Each leaf in the tree represents one gene. The tree branches constitute 12 modules labeled with different colors. (D) Relationships between the coexpressed modules and traits.

WGCNA implements a clustering algorithm for constructing gene coexpression networks on the basis of differential expression data. Therefore, to investigate whether transcriptional regulatory modules are associated with nutrient-deficiency stress in tomato, all 8553 DEGs were used to construct a coexpression network. The weight value β = 16 (*R^2^* = 0.80) was selected (Fig. 5A). According to the expression profile, samples from different tissue sites were clearly separated (Fig. 5B). Twelve valid modules were identified via the dynamic tree cutting method, among which the ‘blue’ module contained the most genes (2632), the ‘sky blue’ module contained the fewest genes (133), and the ‘gray’ module contained 3 genes that were identified as ‘invalid genes’ (Fig. 5C and D). According to the correlations between modules and different stress environments, we identified 3 modules that were significantly correlated (*P* < 0.05) with the nutrient-deficiency response, including the ‘lightcyan’ module (*r* = 0.74, *P* = 0.04), which was positively correlated with LK stress, and the ‘white’ and ‘black’ modules (*r* = −0.85, *P* = 0.008; *r* = −0.78, *P* = 0.02), which were significantly negatively correlated with LP stress. KEGG and GO analyses of genes in the ‘lightcyan’, ‘white’, and ‘black’ modules revealed that these genes are significantly enriched in metabolic pathways such as hormone metabolic process, regulation of the jasmonic acid-mediated signaling pathway, carbon metabolism, amino acid biosynthesis, spliceosome and ribosome biogenesis in eukaryotes (Supplementary Fig. S39). Cytoscape was used to filter out the top 10 hub genes with the highest weights in the ‘lightcyan’ module and construct a gene network map (Supplementary Fig. S40). Among these hub genes, the carbonic anhydrase *SlCA3* (*Solyc02g067750*) can promote carbon dioxide conversion and plant photosynthesis, suggesting that photosynthesis plays an important role in the response of tomato seedlings to low-K stress [55].

### Candidate gene screening via the integration of GWAS and RNA-Seq

To narrow the list of genes related to LN, LP, and LK responses in tomato, we integrated the results from the GWAS and RNA-Seq analysis. A total of 950 DEGs were obtained from the GWAS-associated regions (Supplementary Table S10). Among these, the numbers of DEGs under LN, LP, and LK stress conditions were 420, 737, and 604, respectively. A total of 281 genes were shared among all the datasets, which were located at 81 different loci (Supplementary Fig. S41, Supplementary Table S11). These genes were analyzed by considering their annotations, functional homologs in *Arabidopsis*, and proximity to peak SNPs. By applying this approach, we obtained a list of reasonable candidate genes associated with growth and development under CK and LN, LP, and LK stress conditions. This list included a total of 28 genes that overlap with or are adjacent to significant SNPs and are distributed across 17 key loci (Table 1). Among them, SL2.50chr06_42856514 is a genomic locus that has been reported to be significantly related to leaf nitrogen content under low-nitrogen conditions, whereas the response of other loci to low nitrogen, low phosphorus, and low potassium conditions has not yet been clearly characterized [35].

**Table 1:** Candidate genes selected by GWAS and transcriptome analysis.

**Trait name**	**SNP**	***P-*value**	**Candidate genes**	CK_A_VS_LN_A	CK_B_VS_LN_B	CK_A_VS_LP_A	CK_B_VS_LP_B	CK_A_VS_LK_A	CK_B_VS_LK_B	**Annotation**	***Arabidopsis thaliana* homologous gene**	**ref**
LN_BDW	SL2.50chr01_83983810	5.02 × 10^−8^	Solyc01g090300	NA	−1.17	3.28	NA	2.86	1.23	ERF1b	AT2G44840	[42]
LP_BFW	SL2.50chr01_90191440	2.80 × 10^−6^	Solyc01g099870	1.53	1.07	2.16	1.28	2.06	NA	RAG1-activating protein	AT4G15920	
LP_BDW	SL2.50chr01_90194246	6.05 × 10^−7^	Solyc01g099880	2.08	NA	1.03	NA	2.32	NA	RAG1-activating protein	AT4G15920	
LP_BDW	SL2.50chr01_90194246	6.05 × 10^−7^	Solyc01g100240	NA	1.56	NA	1.97	NA	1.45	Calmodulin-binding protein	AT2G18750	
LP_BFW	SL2.50chr01_90730705	8.09 × 10^−7^	Solyc01g100570	NA	−1.18	NA	−1.83	−1.10	−1.28	Nucleolar protein	AT1G56110	
LP_BFW	SL2.50chr01_90730705	8.09 × 10^−7^	Solyc01g101070	NA	1.28	NA	1.68	NA	1.73	ABC transporter	AT3G53480	
**CK_*CAR***	**SL2.50chr02_42679902**	**1.40 × 10** ^**−7**^	**Solyc02g077880**	**−1.15**	**NA**	**−1.20**	**NA**	**NA**	**NA**	**Auxin-repressed protein**	**AT1G28330**	
LK_AFW	SL2.50chr03_1960858	1.22 × 10^−8^	Solyc03g007460	NA	−2.18	NA	−1.79	NA	−1.07	ERF4	AT4G27950	[42]
LP_BFW	SL2.50chr03_54456471	7.41 × 10^−7^	Solyc03g093140	1.82	NA	1.50	1.09	1.97	1.38	Major facilitator superfamily protein	AT3G47420	
LP_BFW	SL2.50chr03_54456471	7.41 × 10^−7^	Solyc03g093390	−1.75	1.36	NA	2.48	−2.84	1.30	Expansin protein	AT1G65680	
**LP_AFW**	**SL2.50chr04_424610**	**2.35 × 10** ^**−7**^	**Solyc04g005560**	**NA**	**NA**	**−1.08**	**0.89**	**NA**	**NA**	**GEFs**	**AT1G13980**	
LN_*Chla*	SL2.50chr04_1529535	1.42 × 10^−6^	Solyc04g007690	1.40	NA	1.41	NA	1.35	NA	Auxin efflux carrier	AT1G70940	
LN_*Chla*	SL2.50chr04_42397567	1.68 × 10^−6^	Solyc04g008820	NA	−1.59	NA	−1.92	NA	−2.26	HMG TF	AT5G23420	
LN_*CAR*	SL2.50chr06_42856514	3.28 × 10^−7^	Solyc06g069730	3.14	NA	3.47	NA	2.86	NA	Chlorophyll a-b binding protein	AT3G47470	[35]
LN_*Chlb*	SL2.50chr06_43301641	3.28 × 10^−7^	Solyc06g048570	NA	1.06	1.51	2.27	1.01	1.64	Hydrolase alpha/beta fold family protein	AT3G10870	
LN_*Chl(a + b)*	SL2.50chr06_43301655	3.28 × 10^−7^	Solyc06g069600	2.73	NA	3.77	NA	3.26	NA	TF EB	AT4G00050	
LN_*Chl(a + b)*	SL2.50chr06_43301655	3.28 × 10^−7^	Solyc06g069790	2.22	NA	3.03	NA	2.31	NA	Gibberellin-regulated protein	AT5G15230	
LK_PH	SL2.50chr08_64240246	2.17 × 10^−6^	Solyc08g081190	1.10	1.38	NA	1.54	1.13	1.17	Aquaporin 1	AT4G00430	
LK_BFW	SL2.50chr08_878510	1.01 × 10^−6^	Solyc08g006150	NA	−2.13	1.34	NA	NA	−1.25	ChaC cation transport regulator-like 1	AT5G26220	
CK_ADW	SL2.50chr09_69714038	4.21 × 10^−7^	Solyc09g090070	−1.51	1.08	−1.88	1.74	−2.19	1.65	Inorganic phosphate transporter	AT2G38940	[52]
CK_ADW	SL2.50chr09_69714038	4.21 × 10^−7^	Solyc09g090080	−1.17	1.93	−1.79	1.49	−2.69	2.16	Inorganic phosphate transporter	AT2G38940	[52]
CK_ADW	SL2.50chr09_69714038	4.21 × 10^−7^	Solyc09g089930	1.75	NA	2.23	NA	3.89	1.23	ERF1a	AT3G23240	
LP_BDW	SL2.50chr11_1152486	9.07 × 10^−7^	Solyc11g006350	−1.04	−1.40	NA	−2.07	−1.11	−1.50	Aspartate carbamoyl transferase	AT3G20330	
CK_BFW	SL2.50chr11_5253541	2.07 × 10^−9^	Solyc11g012360	2.32	1.29	1.87	2.20	2.57	1.47	Na + −dependent dicarboxylate transporter	AT5G47560	
LN_ADW	SL2.50chr12_2353300	2.65 × 10^−6^	Solyc12g008830	3.63	NA	3.82	NA	2.54	NA	GATA TF	AT4G26150	
LN_PH	SL2.50chr12_353321	3.52 × 10^−7^	Solyc12g005660	3.51	NA	3.71	NA	3.53	NA	CONSTANS-like protein	AT4G27310	
LN_PH	SL2.50chr12_353321	3.52 × 10^−7^	Solyc12g005340	1.07	1.01	1.58	1.65	2.73	2.31	GRAS	AT5G48150	[54]
LN_PH	SL2.50chr12_353321	3.52 × 10^−7^	Solyc12g005310	2.31	NA	1.01	−1.02	1.71	NA	Auxin-responsive GH3-like	AT5G54510	

a The two candidate genes for haplotype analysis are represented in bold.

b Position in base pairs for the lead SNP according to version SL2.50 of the tomato reference sequence.

**Figure 6:**
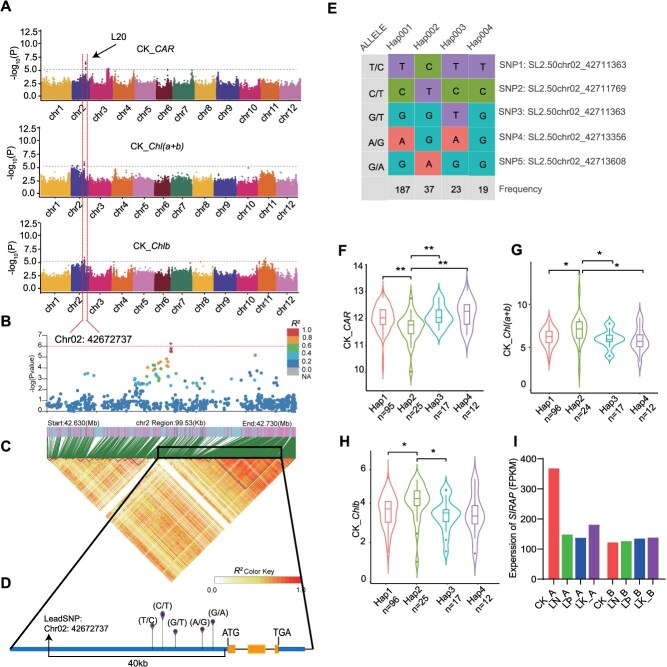
Genome-wide association and transcriptome studies of *SlARP*. (A) Manhattan plot displaying the genome-wide association signals for *CAR* (upper), *Chl*(*a + b*) (middle), and *Chlb* (lower) in the tomato genome. The *y*-axis indicates–log_10_-transformed observed *P*-values. The horizontal dashed lines indicate a genome-wide suggestive threshold (3.91 × 10^−6^). The arrow indicates the most significant signal (L20) on chromosome 2. (B) The genome-wide association signal for L20 is shown in the 42.63–42.73 Mb region (*x*-axis) of chromosome 2. The lead SNP is indicated in square shapes, and the color of each plot corresponds to the *R^2^* value (a measure of LD) according to the legend. (C) Representation of the pairwise *R^2^* values (a measure of LD) among all polymorphic sites in the 99.53-kb genomic region. (D) Gene structure of *SlARP* (*Solyc02g077880*); the yellow box, blue lines, and black lines represent the CDS, promoter and 3′-UTR, and introns, respectively, with the start codon (ATG) and stop codon (TGA) also indicated in the figure. The position of the lead SNP relative to the candidate is indicated. (E) Natural variation in the *SlARP* sequence in 427 diverse tomato accessions. (F–H) Effects of *SlRAP* sequence variation on (F) *CAR* content, (G) *Chl*(*a + b*) content and (H) *Chlb* content in the tomato population. (I) Expression of *SlRAP* in above-ground tissues and below-ground tissues under CK, LN, LP, and LK conditions.

For *CAR*, *Chlb*, and *Chl*(*a + b*) under full nutrition, a common association signal on chromosome 2, L20, was identified with three nearly lead SNPs (SL2.50chr02_42679902, SL2.50chr02_42679782 and SL2.50chr02_42672737) (Fig. 6A). LD analysis between the lead SNP and its neighboring SNPs revealed that there were 51 candidate genes within a 200 kb region centered around the three lead SNPs, including *Solyc02g077880* (encoding auxin-repressed protein, *SlARP*) (Fig. 6B and C). *SlARP* is located 40 kb from the lead SNP

(Fig. 6D), which has previously been reported to be associated with tomato fruit hardness phenotypes [56]. We analyzed allelic variations in *SlARP* using full-length coding sequences (CDS) from 427 resequenced tomato accessions. The five single nucleotide polymorphisms (SNP1-SNP5) detected in the promoter classified *SlARP* into four haplotypes (Fig. 6E). We analyzed the pigment accumulation of these four haplotype samples and found that the accessions with haplotype 2 accumulated significantly less *CAR* and more *Chlb* and *Chl*(*a + b*) than the other haplotypes did (Fig. 6G and H, *P* < 0.05). Combining haplotype analysis and pigment content analysis, we found that the SNPs SL2.50chr02_42711363 (SNP1), SL2.50chr02_42711769 (SNP2), and SL2.50chr02_42713608 (SNP5) may affect gene function. The RNA-Seq results revealed that, compared with that under CK, the expression of *SlARP* decreased in above-ground tissues under LN, LP, and LK stress, whereas there was no difference in the expression of *SlARP* in below-ground tissues under LN, LP, and LK stress (Fig. 6I). These results further suggest that *SlAPR* may be involved in the synthesis of *CAR* and *Chlb* in tomato leaves.

**Figure 7:**
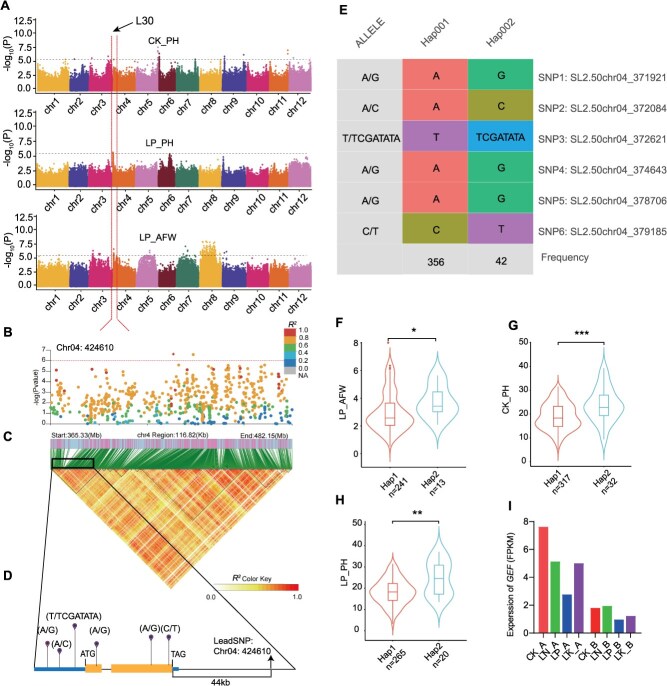
Genome-wide association and transcriptome study of *GEF*. (A) Manhattan plot displaying the genome-wide association signals for CK_PH (upper), LP_PH (middle) and LP_AFW (lower) in the tomato genome. (B) The arrow indicates the most significant signal (L30) on chromosome 4. The genome-wide association signal for L30 is shown on the 356.33–482.15 Mb region (*x*-axis) of chromosome 4. The lead SNP is indicated in square shapes, and the color of each plot corresponds to the *R^2^* value (a measure of LD) according to the legend. (C) Representation of the pairwise *R^2^* values (a measure of LD) among all polymorphic sites in the 116.82-kb genomic region. (D) Gene structure of *GEF* (*Solyc04g005560*); the yellow box, blue lines, and black lines represent the CDS, promoter and 3′-UTR, and introns, respectively, with the start codon (ATG) and stop codon (TGA) also indicated in the figure. The position of the lead SNP relative to the candidate is indicated. (E) Natural variation in the *GEF* (*Solyc04g005560*) sequence in 427 diverse tomato accessions. (F–H) Effects of *GEF* sequence variation on (F) AFW under LP stress, (G-H) PH under CK conditions and LP stress in the tomato population. (I) Gene expression of *GEF* in above-ground tissues and below-ground tissues under the CK, LN, LP, and LK conditions.

Interestingly, after LP conditions, the GWAS of LP_PH, LP_AFW and CK_PH revealed another common association signal on chromosome 4, L30, with three nearly lead SNPs (SL2.50chr04_319830, with a *P*-value of 2.99 × 10^−6^; SL2.50chr04_352448, with a *P*-value of 1.99 × 10^–6;^ and SL2.50chr04_424610, with a *P*-value of 2.35 × 10^−7^) (Fig. 7A and B; Supplementary Table S4). LD analysis between the lead SNP and its neighboring SNPs revealed 77 candidate genes, including *Solyc04g005560*, which is annotated as guanine nucleotide exchange factor-like protein (*GEF*) (Fig. 7C). We analyzed allelic variations in *GEF* using full-length CDS from 427 resequenced tomato accessions (Fig. 7D). Three SNPs were detected in the CDS region, whereas 2 SNPs and 1 indel were identified in the promoter region, dividing *GEF* into two haplotypes (Fig. 7E). The two haplotypes were analyzed for their performance under LP stress in terms of PH, above-ground tissue fresh weight (AFW), and PH under CK conditions. The results revealed that under both LP stress and CK conditions, haplotype 2 presented significantly greater PH and AFW than did haplotype 1 (Fig. 7F–H). These observations indicate that the two haplotypes respond differently to environmental stress, thereby affecting plant growth in distinct ways. Further analysis revealed that the three SNPs located in the CDS region were all nonsynonymous mutations. Notably, the Indel (T/TTCGATATA at position −1760) in the promoter may affect its activity. The RNA-Seq results revealed that the expression of *GEF* decreased in the above-ground tissues and below-ground tissues under LP and LK stress and that the expression of *GEF* in the above-ground tissues decreased but remained stable in the below-ground tissues under LN stress (Fig. 7I). These results suggest that *GEF* is a potential gene involved in the response to low-Pi stress.

## Discussion

N, P, and K are macronutrients that are essential for plant growth and development, and their deficiency can affect plant morphology and physiological activities. Investigating the molecular mechanisms underlying plant responses to LN, LP, and LK can provide insights into farmland fertilizer management strategies, enhancing nutrient utilization efficiency and ultimately increasing crop yields and quality. This study systematically analyzed phenotypic variation across nine traits in 427 accessions under CK, LN, LP, and LK conditions. Notable differences were observed in the responses of the three major tomato subgroups to LN, LP, and LK stress. Notably, the SPIM subgroup demonstrated superior adaptability, potentially due to its unique genetic background and ecological adaptability, and these factors collectively enable the subgroup to exhibit increased nutrient absorption and utilization abilities [57]. To investigate the molecular mechanisms behind these phenotypic variations, we conducted a GWAS on 28 phenotypic datasets collected under LN, LP LK, and CK conditions and analyzed DEGs in below-ground tissues and above-ground tissues under these same conditions. By integrating the results of GWAS and transcriptome analysis, a total of 20 significant SNPs were identified, corresponding to 17 genetically significant loci and 28 candidate genes, providing a genetic basis for the breeding of tomato to optimize efficient nutrient utilization (Table 1).

According to optimal allocation theory [58], plants allocate resources among different organs (such as roots, stems, and leaves) on the basis of environmental conditions (such as light, nutrients, and water) to maximize their adaptability. Under the stress of low N, P, and K, the biomass of the below-ground part of tomato plants increases to increase nutrient absorption, and the growth of the above-ground part decreases to reduce energy consumption. This strategy may be driven by the differential expression of root development-related genes such as *Solyc04g005560*, whose *Arabidopsis* homolog *AT1G13980* has been shown to influence lateral root formation by regulating auxin distribution [44, 45]. Low nitrogen affects the content of photosynthetic pigments in tomato leaves, resulting in leaf yellowing [59]. However, the underlying reasons for this change are still unclear. Our experimental results revealed that the yellowing of leaves may be caused by a decrease in the *Chlb* content and an increase in the carotenoid content (Fig. 2). *Chlb* plays a role in the carbon sequestration stage of photosynthesis and is easily affected by changes in nutritional status, and an increase in carotenoids may alleviate the photooxidative damage induced by nitrogen-deficiency through a photoprotective mechanism [60, 61].

GWAS serves as a powerful tool for pinpointing genetic variants linked to complex genetic traits, and it has been widely applied in the research of diverse crops, such as rice, maize, peach, watermelon, and tomato [62–66]. In our study, a total of 116 loci were identified by GWAS from 28 datasets, of which 89 loci were actually mapped under LN/LP/LK conditions, including 6 loci that have been reported to be involved in the LN/LP/LK response [36–38]. For example, L58 was simultaneously detected in LN_*CAR*, LN_*Chla*, LN_*Chlb* and LN_*Chl*(*a + b*), and L109 and L110 were detected in LN_AFW and LN_BDW. L58, L109 and L110 overlapped with the locus previously reported to respond to low nitrogen stress [35], supporting the accuracy of our GWAS results. In addition, 9 loci were simultaneously associated with different deficiency conditions, and many candidate genes, including *chlorophyll a-b binding protein* and *inorganic phosphate transporter*, were identified. Notably, among the 116 significantly associated loci identified, the CK_PH trait exhibited the greatest number of associated loci, totaling 13. This suggests that the genetic variation underlying PH in tomato is more diverse and thus more readily detectable through GWAS [37].

In our research, we identified DEGs in above-ground tissues and below-ground tissues after LN, LP and LK treatment by RNA-Seq. Notably, the number of DEGs in below-ground tissues (2644, 4475, and 3846) exceeded that in above-ground tissues (1724, 3027, and 2280) under all three stress conditions. This disparity may be due to the direct involvement of below-ground tissues in nutrient absorption, making them more responsive to environmental stressors. Thousands of genes exhibited varied expression patterns under different stress conditions, reflecting the intricate response mechanisms of tomato to LN, LP, and LK stress. For example, chlorophyll a-b binding protein (*AT3G47470*) has been reported to be involved in the process of converting light and chemical energy during photosynthesis in *Arabidopsis thaliana* [67], and its homologous gene was upregulated in the above-ground tissues under all three stress conditions. Additionally, the expression of a phosphate kinase (*Solyc09g008480*) and an inorganic phosphate transporter (*Solyc09g090070*) in the below-ground tissues was upregulated, which is consistent with previous research on the response of rice roots to phosphorus [68]. Moreover, the 103 core DEGs showed a high degree of conservation with deficient nutrient response genes in other species, including nitrate transporters, the MYB TF family, the TCP TF family, and other genes associated with plant nutrient absorption and transport [69–71]. These findings suggest that the regulatory pathways involved in the response of tomato to nutrient-deficiency differ between the above-ground and below-ground parts and that the response mechanisms of tomato and other crops may be conserved.

To locate nutrient-deficiency response genes precisely, we integrated the candidate genes associated with GWASs and the DEGs identified under three stress conditions from the transcriptome. We identified 3302 genes by GWAS and 2719 genes by RNA-Seq, and the number of candidate genes was reduced to 950 after integration, representing a 118-fold reduction in the range of candidates. Interestingly, 281 genes were common across the LN, LP, and LK stress conditions, including 28 high-availability genes that may play a role in the deficiency response (Table 1). For example, some TFs from the GRAS family have been reported to participate in plant abiotic stress responses [72], such as known inorganic phosphate transporters [69], and plant hormone response proteins [73]. These results suggest that the response of tomato to LN, LP, and LK deficiency is highly complex and that the method of integrating GWAS with transcriptome analysis holds great potential.

Our research provides novel insights into the genetic mechanisms by which tomatoes respond to LN, LP, and LK stress through a comprehensive analysis combining GWAS and RNA-Seq. Several candidate genes and key loci associated with LN, LP, and LK stress have been identified, offering rich resources for the genetic improvement of tomato. These genes and the possible causative SNPs can serve as potential targets for increasing nutrient utilization efficiency, ultimately increasing crop yields and quality.

## Materials and methods

### Plant materials and experimental design

A previous study has detailed the history of tomato domestication and improvement on the basis of a classical population of 427 tomato accessions [33, 40]. A globally diverse collection of 427 tomato accessions, comprising 31 *Solanum pimpinellifolium* accessions (SPIM = 31), 172 *S. lycopersicum* var. *cerasiforme* accessions (SLC = 172) and 224 *S. lycopersicum* accessions (SLL = 224), was used for phenotypic and genotypic surveys, as well as GWAS analysis.

The 427 tomato accessions were grown in a smart greenhouse at the Vegetable Base of Huazhong Agricultural University, which is located in Wuhan, China. The germinated seeds were transferred into 50-cell trays filled with a 1:1:1 soil-peat-perlite substrate mixture during mid-September 2022. After 14 days in the trays, the seedlings were moved to four distinct nutrient conditions within environments that contained only vermiculite. The greenhouse was equipped with ventilation, temperature, and humidity control systems.

The plants were irrigated using hydroponic methods with equal volumes of nutrient solutions to ensure different treatments. The micronutrient and iron compositions of the solutions were 11.6 μM H_3_BO_3_, 2.4 μM MnSO_4_·H_2_O, 0.2 μM ZnSO_4_·7H_2_O, 0.1 μM CuSO_4_·5H_2_O, 0.1 μM NaMoO_4_·2H_2_O, 50 μM FeSO_4_·7H_2_O, and 50 μM EDTA-Na_2_. The compositions of the nutrient solutions for the different treatments were as follows:

- CK (full nutrients): (0.8 mM Ca(NO_3_)_2_·4H_2_O, 1.5 mM KNO_3_, 0.75 mM MgSO_4_·7H_2_O, and 0.83 mM K_2_HPO_4_·3H_2_O)
- LN (low nitrogen): (0.8 mM CaCl_2_, 1.35 mM KCl, 0.15 mM KNO_3_, 0.75 mM MgSO_4_·7H_2_O, and 0.83 mM K_2_HPO_4_·3H_2_O)
- LP (low phosphorus): (0.8 mM Ca(NO_3_)_2_·4H_2_O, 1.5 mM KNO_3_, 1.73 mM KCl, 0.75 mM MgSO_4_·7H2O, and 0.05 mM K_2_HPO_4_·3H_2_O)
- LK (low potassium): (0.8 mM Ca(NO_3_)_2_·4H_2_O, 0.83 mM Na_2_HPO_4_·12H_2_O, 1.35 mM NaNO_3_, 0.75 mM MgSO_4_·7H_2_O, and 0.15 mM KNO_3_)

The pH of the nutrient solutions was adjusted to 5.5 using 1 mM HCl and NaOH [74]. The nutrient mixture was replenished every 5 days. The tomato plants were maintained in nutrient mixture for approximately 2 weeks. For the experiment, 50-cell trays were used for spaced planting, with 5 replicates per variety and 5 varieties per tray. Field management followed standard agricultural practices.

The 28 phenotypic datasets were classified into three categories: 4 traits related to length, 16 traits related to quality, and 8 traits related to pigment content. Agronomic traits were measured in plants with consistent growth, with three biological replicates for each accession. PH was measured from the stem base to the apical growth point using a ruler. The other above-ground traits were weighed using an analytical balance. For below-ground traits, root systems were collected from below the base of the stem. The soil was first removed without damaging the roots, and the substrate residues were then removed by gently shaking the roots. Dry fresh samples at 80°C to constant weight. After drying, the above-ground and below-ground dry weights were measured using an analytical balance. To analyze chlorophyll-related traits, 0.1 g of fresh leaf tissue was powdered and extracted with 1 ml of 95% ethanol in the dark for 4 hours. The supernatant was added to an ELISA plate, and each sample was analyzed in triplicate. The absorbance values were read at 645 nm, 663 nm, and 470 nm via a microplate reader to calculate the contents of chlorophyll and carotenoids. All the data were carefully reviewed and curated before analysis.

### Phenotypic data processing and statistical analysis

Statistical processing was conducted with Microsoft Excel and R software v3.6.2. Specifically, phenotypic value distributions across agronomic traits were assessed through frequency analysis in the spreadsheet application. Variability quantification employed the CV, mathematically defined as the ratio of standard deviation (σ) to arithmetic mean (μ), with μ corresponding to the population mean within the genetic association panel. To assess the significance of differences in agronomic traits across the four treatments (CK, LN, LP, and LK), a *t* test was conducted. An analysis of variance was performed to evaluate the differences among the three subgroups (SPIM, SLC, and SLL). To explore significant differences between the pairwise means of the subgroups, Tukey’s HSD test was applied. The correlations between variables were analyzed using the Corrplot package in R (v3.6.2), and the results were visualized via a correlogram.

### Population structure and kinship analysis

The genomic sequences of the 427 tomato accessions are accessible through the National Center for Biotechnology Information Sequence Read Archive, with accession numbers SRP045767 and PRJNA666021. The BWA software [75] was implemented to align all paired-end sequence reads to the Heinz 1706 tomato reference genome (SL2.5 v75), and SNPs were subsequently identified with the Genome Analysis Toolkit [76].

The genotyping-by-sequencing variant dataset was screened to preserve SNPS with a minor allele frequency (MAF) of at least 5% and a deletion rate of less than 20%. This resulted in a final dataset of 322 580 SNPs from 427 tomato accessions for further analysis. PCA was conducted using PLINK version 1.9 [77], and the emmax-kin program from EMMAX was implemented to analysis kinship matrix [78].

### Genome-wide association study

The GWAS was performed on SNPs meeting quality control thresholds (MAF ≥0.05, missing data <20%). Phenotypic associations for all 28 traits were evaluated through the EMMAX-implemented mixed linear model approach [78]. We used the first 10 principal components and the first 5 principal components, respectively, as parameters for population correction and the kinship matrix (K) as a covariate to correct population structure. Missing genotypes were inputted via Beagle software [79]. A total of 322 580 independent valid SNPS participated in the genome-wide association analysis, the thresholds for suggestive association and significant association thresholds corresponded to *P* = 1/n (3.11 × 10^−6^) and *P* = (0.05/n) 1.55 × 10^−7^, where n represented the effective number of independent SNPs. The R package qqman was used to visualize the GWAS results, including Manhattan plots and QQ plots. LDBlockShow is used for the calculation and drawing of the LD block [80]. The 138 significant lead SNPs across the tomato genome were grouped into different loci by dividing the genome into 200-kb segments, and the total number of significant loci was calculated. The SNP with the smallest *P*-value within each locus was considered the lead SNP.

### RNA-Seq analysis

The classic cultivated tomato *Ailsa Craig* (AC) was selected as the material and subjected to LN, LP, or LK stress for 24 hours at the seedling stage, after which the aboveground and belowground tissues were collected for total RNA extraction and sequencing. In total, eight samples (two types of tissues × four treatments) were sequenced. RNA sequencing libraries were constructed with the kit (NEB, USA). After pooling qualified libraries, paired-end 150 bp sequencing was conducted using Illumina sequencing platforms at Novogene Corporation. The raw reads were processed to obtain high-quality clean reads by filtering with Fastp [81].

After clean reads were obtained, alignment to the tomato reference genome (Heinz 1706; SL2.5) was performed using HISAT2. Gene expression quantification was performed via HTSeq software, utilizing the fragments per kilobase of exon model (FPKM per million mapped reads) normalization method [82]. GFOLD V1.1.4 was used to identify DEGs between treatments (log2-fold change >1 or < 1) [48]. The functional annotation of the DEGs was carried out using the GO and Kyoto Encyclopedia of Genes and Genomes (KEGG) databases (http://geneontology.org), with the clusterProfiler function in R. The expression patterns of the DEGs were analyzed using the muffz package in R software. [83].

### Weighted gene coexpression network analysis

The weighted gene coexpression network analysis (WGCNA) R package (v1.72) was used to construct gene coexpression networks and explore gene functions across different environments [84]. We conducted a WGCNA on 8553 DEGs identified under at least one treatment condition. The FPKM values of these DEGs were used for WGCNA. To approximate a scale-free topology, a soft-thresholding power of 16 was chosen (model fitting index *R^2^* = 0.8). The biological relevance of the network was examined by assessing the mean connectivity and scale-free topology over the range of soft threshold capability (β). Hierarchical clustering of all CDS was performed using a topological overlap-based dissimilarity measure [85]. Finally, module detection was conducted using the dynamic tree cut method, with parameters set to minModuleSize = 100 and mergeCutHeight = 0.2. The regulatory network of candidate genes was mapped using Cytoscape software [86]. After the significantly correlated modules were identified, GO and KEGG annotations and enrichment analyses were performed for the genes in the modules. Finally, the coexpression network was visualized using Cytoscape (v3.10.1). In each module, nodes with weights >0.3 were selected for gene interaction network construction, the MCC algorithm in the cytoHubba plug-in was used for calculation, and the top 15 genes were used as Hub genes.

### Gene expression analysis

To verify the transcriptome sequencing results, the related expression of *Solyc03g112390* (R2R3MYB TF), *Solyc09g090070* (PHT, inorganic phosphate transporter), and *Solyc01g010480* (*KCNH8*, potassium voltage-gated channel subfamily H member 8) were quantified by qRT–PCR [46, 47]. The primer pairs were listed in Supplementary Table S12. Each sample was subjected to three technical replicates of qRT–PCR using SYBR Green Real Master Mix. The comparative *C*_T_ method was used to quantify the gene expression [87].

### Characterization of candidate genes

A 400-kb genomic region spanning 200-kb upstream and downstream of the significant SNPs was used to screen for candidate genes. These genes were then extracted from tomato reference genome (Heinz 1706; vSL2.5) by BEDTools [88, 89]. To select potential candidate genes for the 28 agronomic traits, we referred Arabidopsis homologous genes and previous reports. GenehapR was used to perform haplotype analysis, utilizing SNPs from the CDS and promoter regions of the gene. [90].

## Supplementary Material

Web_Material_uhaf112

## Data Availability

All data are included in the paper and in the Supplementary Materials published online.
