# Supplementary material for: Integration of GWAS and transcriptome approaches for the identification of nitrogen-, phosphorus-, and potassium-responsive genes in tomato
Source: Hortic Res. 2025 Apr 24;12(7):uhaf112. doi: 10.1093/hr/uhaf112 (PMC12227991; doi:10.1093/hr/uhaf112)
Supplement: Web_Material_uhaf112 [file web_material_uhaf112.zip › Supp Fig.docx]

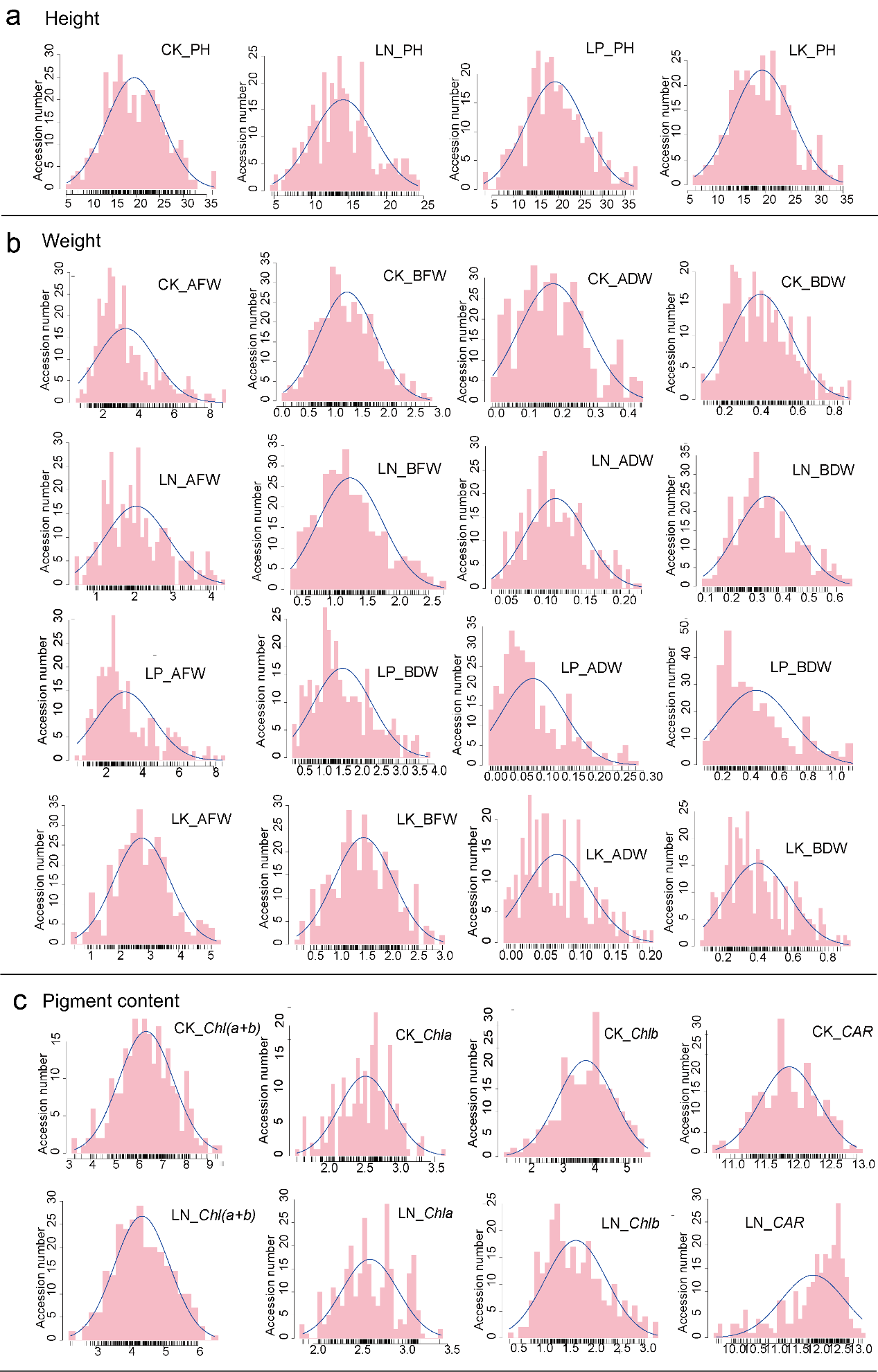


**Supplementary Figure 1: Frequency distribution of 9 phenotypic values in the 427 tomato accessions under full nutrient, low nitrogen, low phosphorus and low potassium.** A total of 28 datasets were satisfied. The 28 datasets were classified into three categories: 4 plant height datasets (**a**), 16 plant weight datasets (**b**) and 8 pigment content datasets (**c**). CK_PH: Plant height (cm) under full nutrient, LN_PH: Plant height (cm) under low nitrogen stress, LP_PH: Plant height (cm) under low phosphorus stress, LK_PH: Plant height (cm) under low potassium stress, CK_AFW: Above-ground tissues fresh weight (g) under full nutrient, LN_AFW: Above-ground tissues fresh weight (g) under low nitrogen stress, LP_AFW: Above-ground tissues fresh weight (g) under low phosphorus stress, LK_AFW: Above-ground tissues fresh weight (g) under low potassium stress, CK_ADW: Above-ground tissues dry weight (g) under full nutrient, LN_ADW : Above-ground tissues dry weight (g) under low nitrogen stress, LP_ADW: Above-ground tissues dry weight (g) under low phosphorus stress, LK_ADW: Above-ground tissues dry weight (g) under low potassium stress, CK_BFW: Below-ground tissues fresh weight (g) under full nutrient, LN_BFW: Below-ground tissues fresh weight (g) under low nitrogen stress, LP_BFW: Below-ground tissues fresh weight (g) under low phosphorus stress, LK_BFW: Below-ground tissues fresh weight (g) under low potassium stress, CK_BDW: Below-ground tissues dry weight (g) under full nutrient, LN_BDW: Below-ground tissues dry weight (g) under low nitrogen stress, LP_BDW: Below-ground tissues dry weight (g) under low phosphorus stress, LK_BDW: Below-ground tissues dry weight (g) under low potassium stress, CK_*Chla*: Chlorophyll *a* content (mg/g) under full nutrient, LN_*Chla*: Chlorophyll *a* under low nitrogen stress (mg/g), CK_*Chlb*: Chlorophyll *b* under full nutrient (mg/g), LN_*Chlb*: Chlorophyll *b* under low nitrogen stress (mg/g), CK_*Chl(a+b)*: Chlorophyll under full nutrient (mg/g), LN_Chl*(a+b)*: Chlorophyll under low nitrogen stress (mg/g), CK_*CAR*: Carotenoids under full nutrient (mg/g),LN_*CAR*: Carotenoids under low nitrogen stress (mg/g).


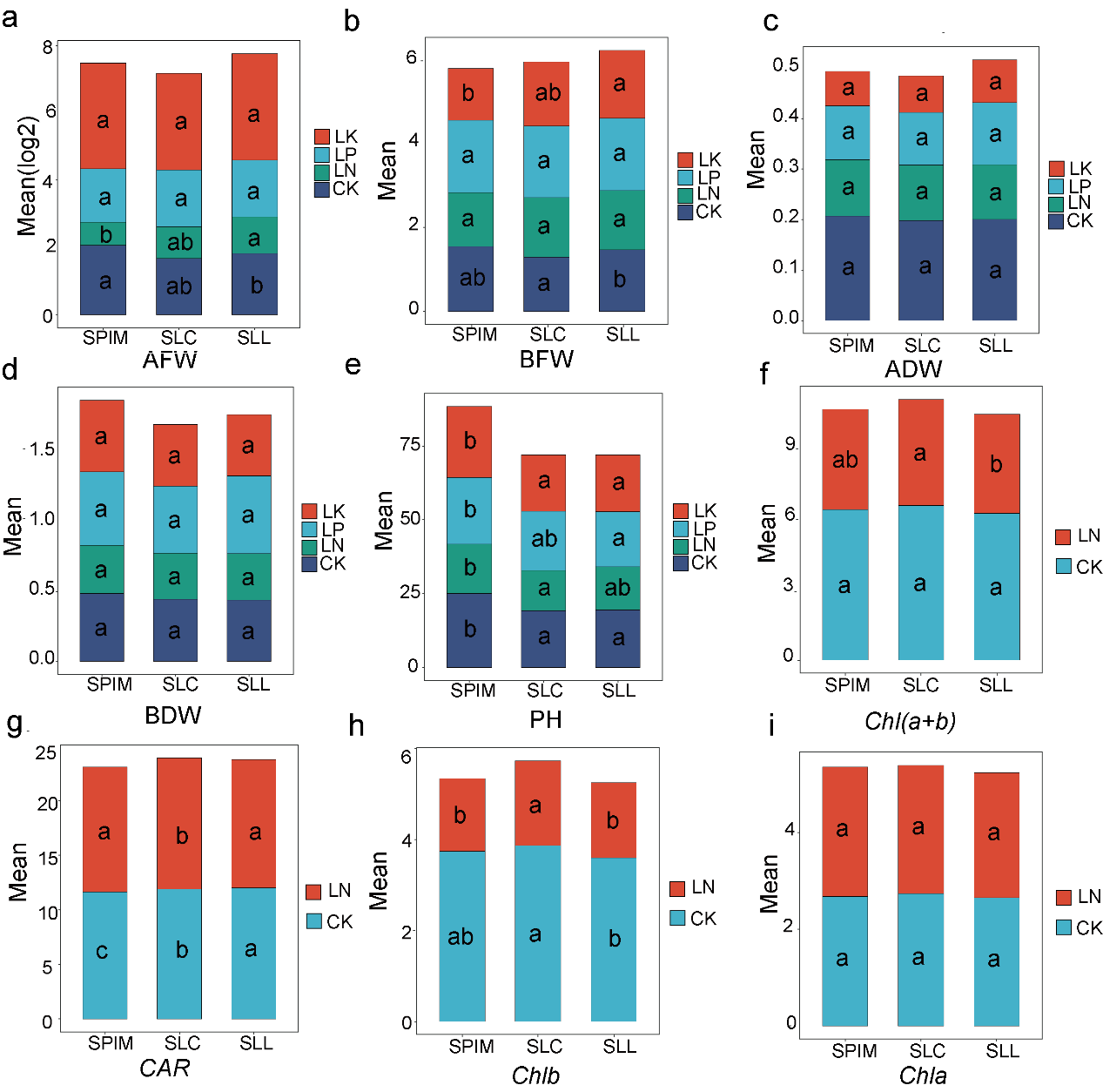


**Supplementary Figure 2: Phenotypic variation of low nitrogen (LN), low phosphorus (LP) and low potassium (LK) responses in the different tomato subgroups.** **(a) AFW:** Above-ground tissues fresh weight (g), **(b)** **BFW:** Below-ground tissues fresh weight (g)**, (c)** **ADW:** Above-ground tissues dry weight(g), **(d) BDW:** Below-ground tissues dry weight(g), **(e) PH:** plant height(cm), **(f) *Chl(a+b)*:** Chlorophyll under full nutrient (mg/g), **(g) *CAR*:** Carotenoids content(mg/g), **(h) *Chlb*:** Chlorophyll *b* content (mg/g), **(i) *Chla*:** Chlorophyll *a* content (mg/g). SPIM, *S. pimpinellifolium*. SLC, *S. lycopersicum* var. *cerasiforme*. SLL, *Solanum lycopersicum* var. *lycopersicum*.


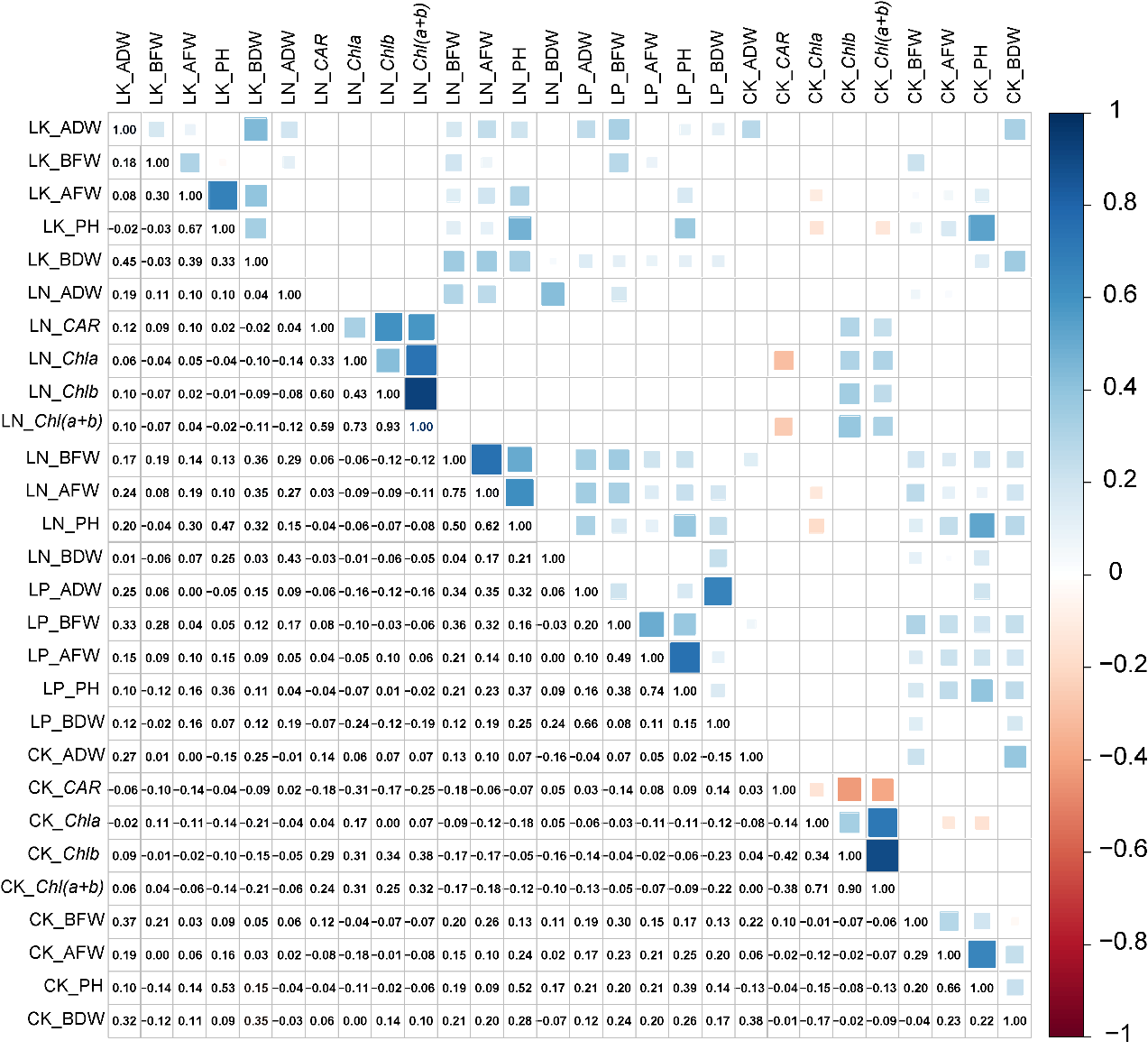


**Supplementary Figure 3:** **Correlations between the analyzed phenotypes.** The correlation coefficient (Spearman) ranges from -1 (red color) to +1 (blue color).


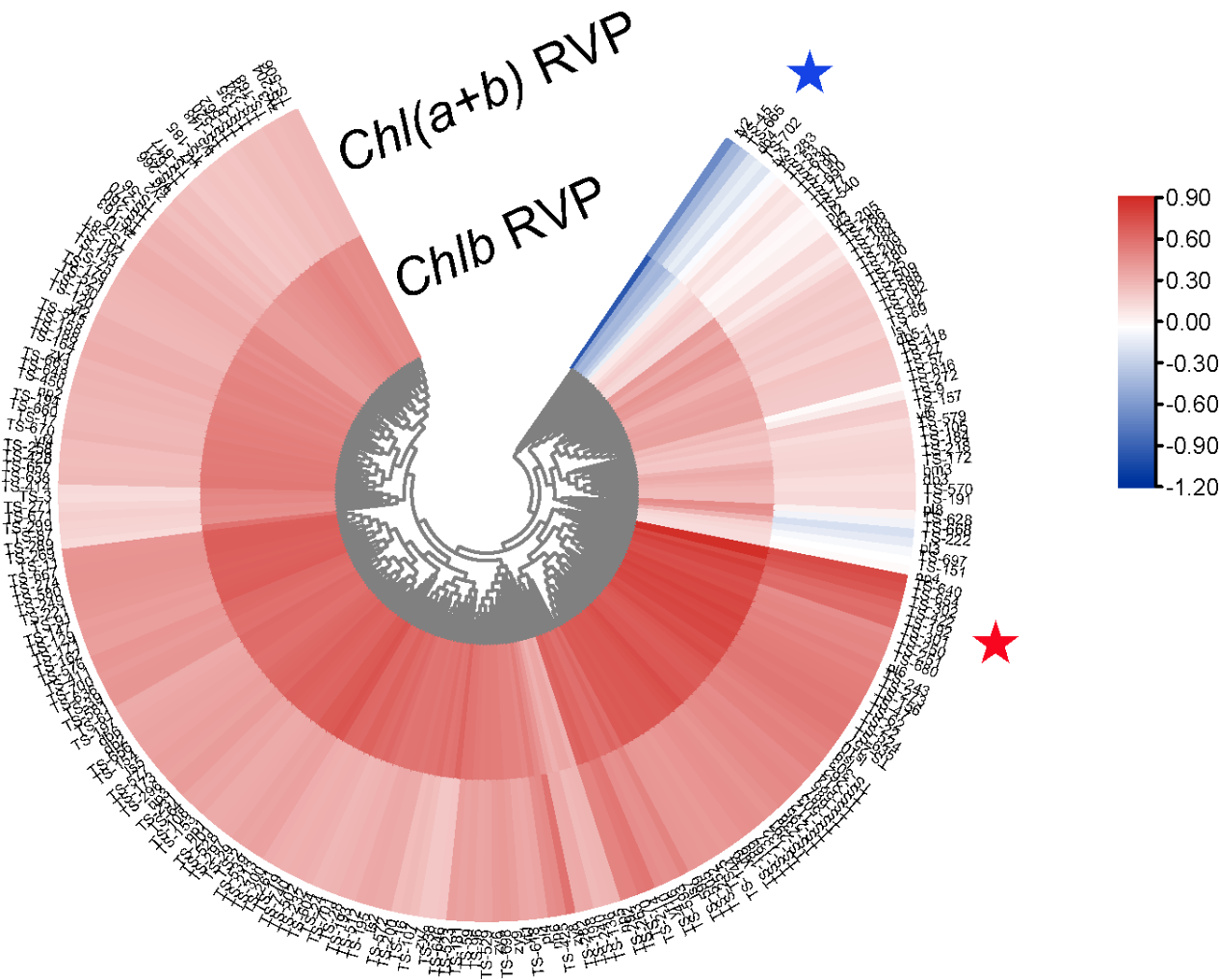


**Supplementary Figure 4: Cluster analysis of relative percentages of chlorophyll (*a+b*) and chlorophyll *b* contents under LN and CK treatments.** *Chl*(*a+b*) RVP, the relative percentage of chlorophyll *b* content under LN and CK treatments. *Chlb* RVP, the relative percentage of chlorophyll *b* content under LN and CK treatments. The relative percentage is calculated by the formula (CK-LN)/CK; “★”, the clustering results of extremely LN sensitive materials; “★”, the clustering results of extremely LN tolerant materials.


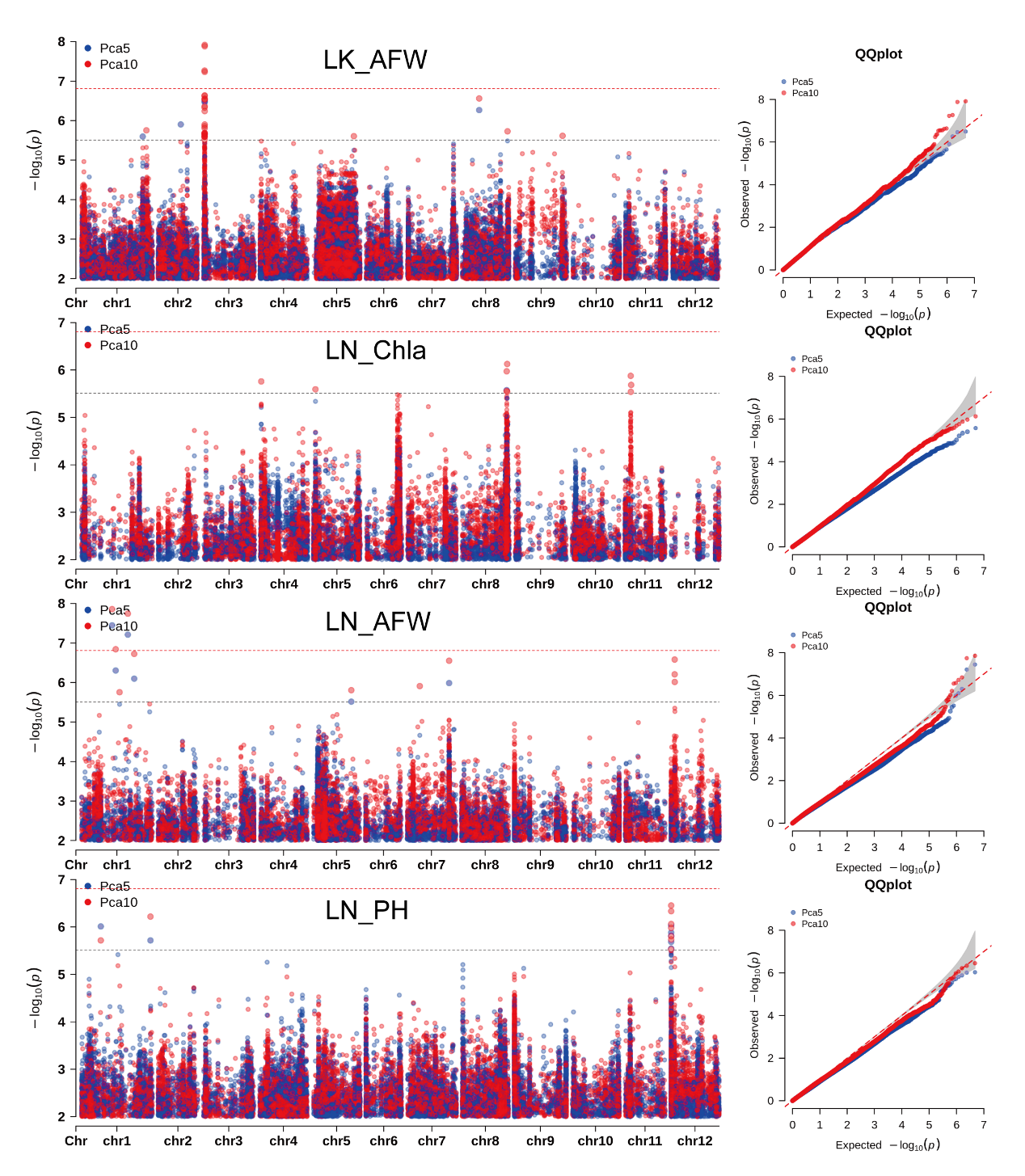


**Supplementary Figure 5: Manhattan and QQ plots generated using two mapping models.** The -log_10_ (*p*) values from the GWAS results are plotted on the *y*-axis. The red line represents the genome-wide significance threshold (1.55×10^-7^), while the black line indicates the suggestive threshold (3.1×10^-6^). In the Manhattan plot, blue represents PCA5 +K, and red represents PCA10 +K. The QQ plots are displayed to the right of their corresponding Manhattan plots.


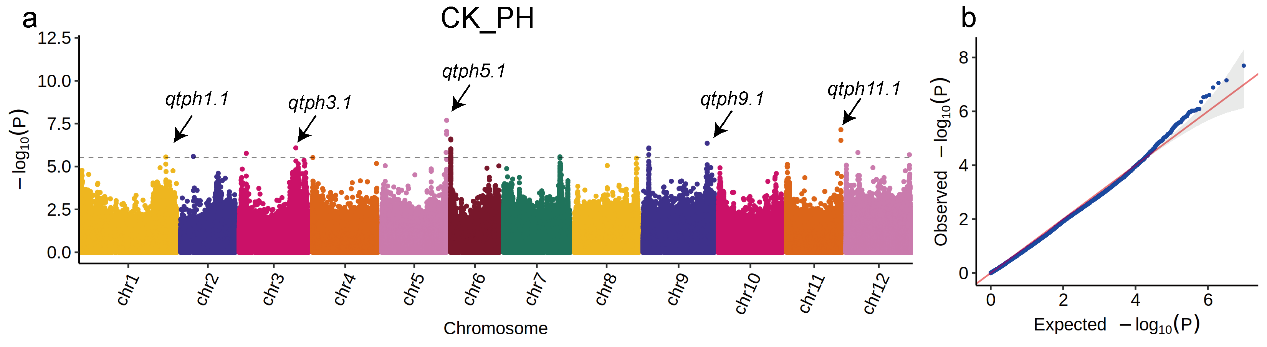


**Supplementary Figure 6:** **Manhattan plot (a) and Quantile-Quantile (Q-Q) plot (b) of GWAS for CK_PH.** Negative log_10_-transformed *P* values from the compressed mixed linear model were plotted against SNPs position on each of the 12 chromosomes. The horizontal dashed line indicates a genome-wide significance threshold of 3.1×10^-6^.


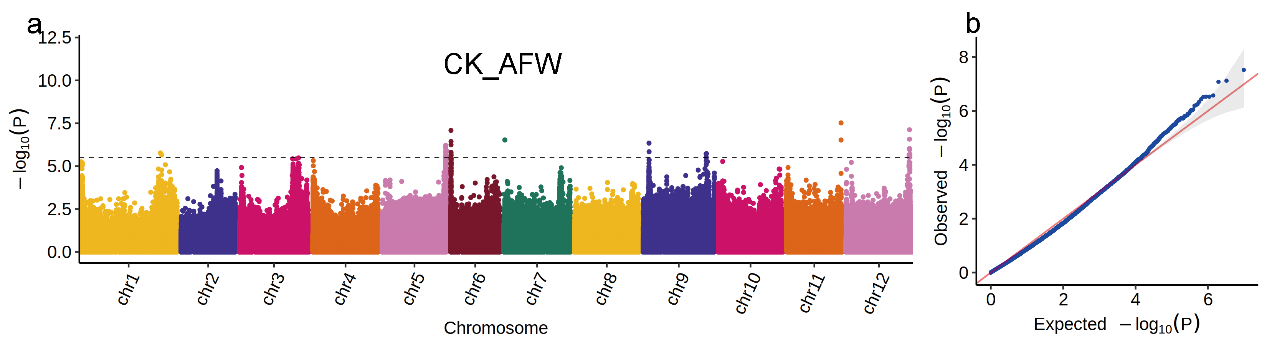


**Supplementary Figure 7: Manhattan plot (a) and Quantile-Quantile (Q-Q) plot (b) of GWAS for CK_AFW.** Negative log_10_-transformed *P* values from the compressed mixed linear model were plotted against SNPs position on each of the 12 chromosomes. The horizontal dashed line indicates a genome-wide significance threshold of 3.1×10^-6^.


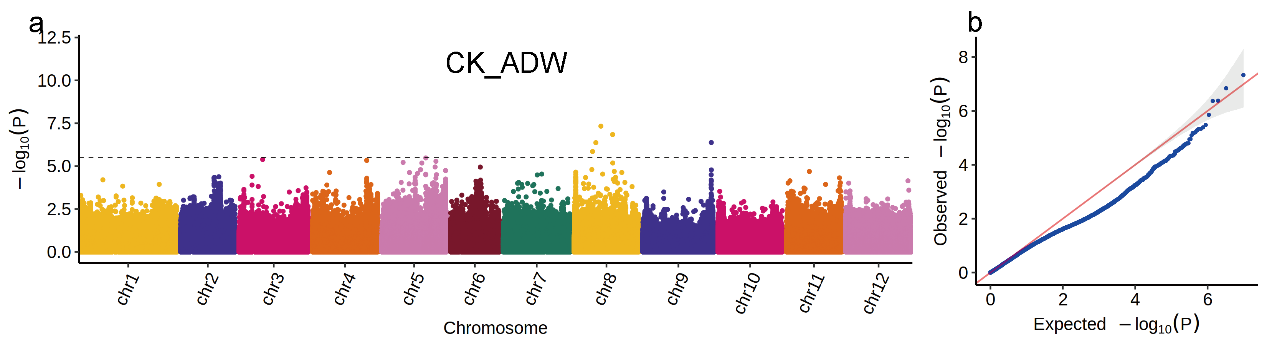


**Supplementary Figure 8: Manhattan plot (a) and Quantile-Quantile (Q-Q) plot (b) of GWAS for CK_ADW.** Negative log_10_-transformed *P* values from the compressed mixed linear model were plotted against SNPs position on each of the 12 chromosomes. The horizontal dashed line indicates a genome-wide significance threshold of 3.1×10^-6^.


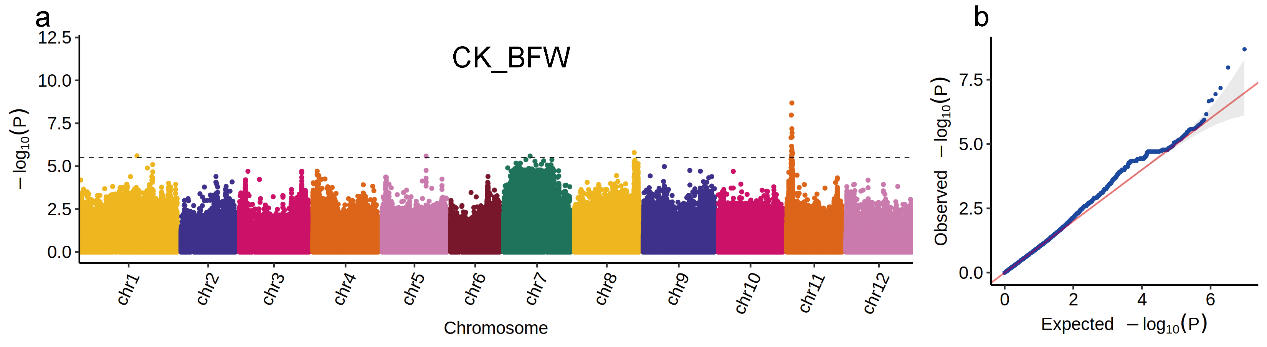


**Supplementary Figure 9: Manhattan plot (a) and Quantile-Quantile (Q-Q) plot (b) of GWAS for CK_BFW.** Negative log_10_-transformed *P* values from the compressed mixed linear model were plotted against SNPs position on each of the 12 chromosomes. The horizontal dashed line indicates a genome-wide significance threshold of 3.1×10^-6^.


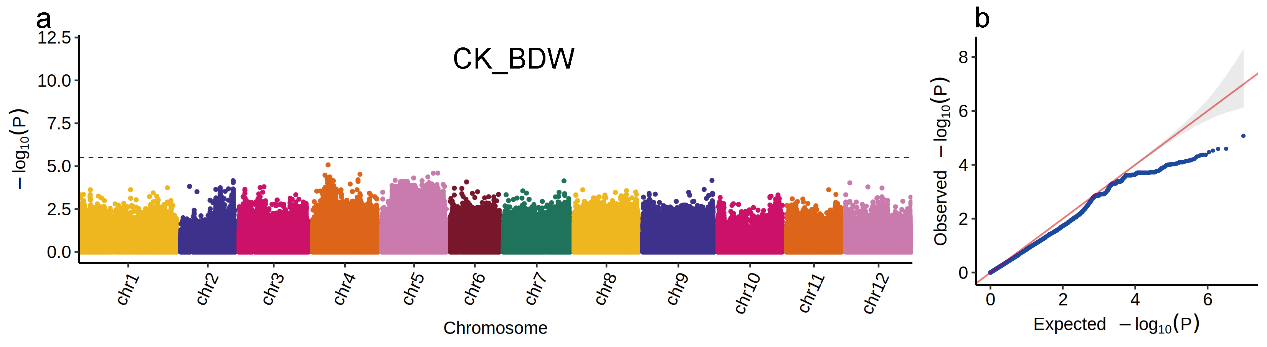


**Supplementary Figure 10: Manhattan plot (a) and Quantile-Quantile (Q-Q) plot (b) of GWAS for CK_BDW.** Negative log_10_-transformed *P* values from the compressed mixed linear model were plotted against SNPs position on each of the 12 chromosomes. The horizontal dashed line indicates a genome-wide significance threshold of 3.1×10^-6^.


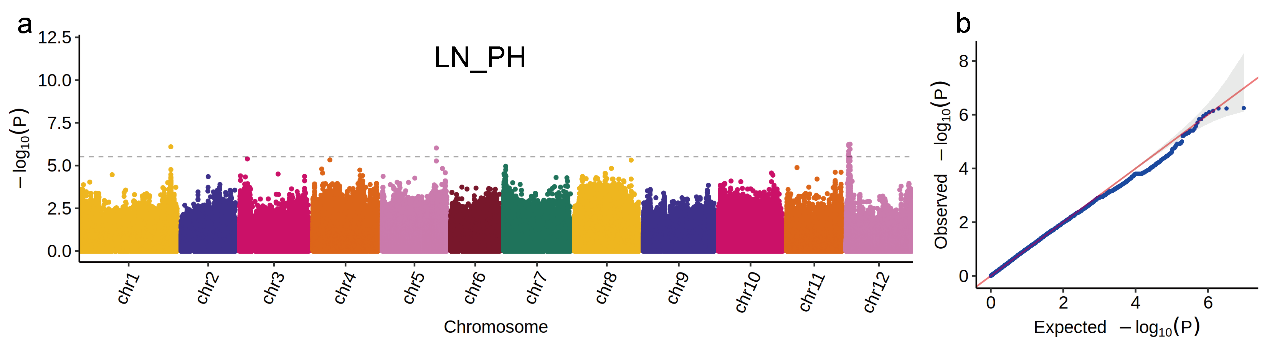


**Supplementary Figure 11: Manhattan plot (a) and Quantile-Quantile (Q-Q) plot (b) of GWAS for LN_PH.** Negative log_10_-transformed *P* values from the compressed mixed linear model were plotted against SNPs position on each of the 12 chromosomes. The horizontal dashed line indicates a genome-wide significance threshold of 3.1×10^-6^.


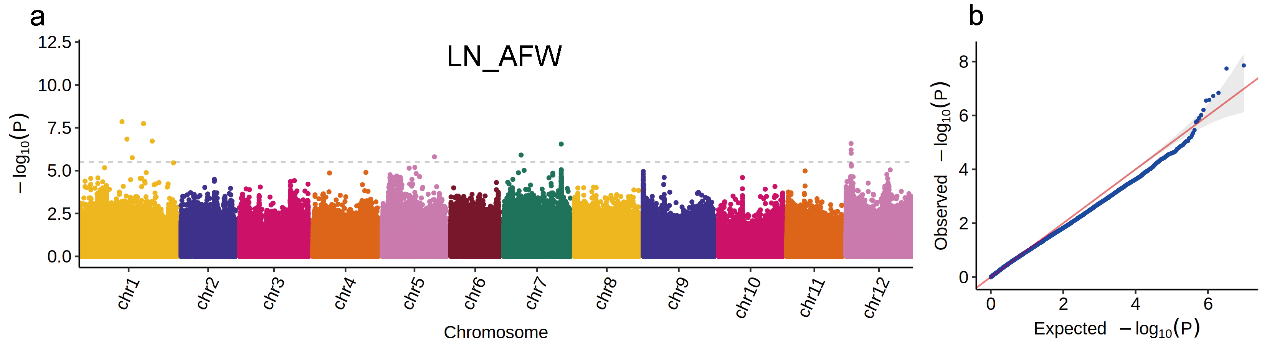


**Supplementary Figure 12: Manhattan plot (a) and Quantile-Quantile (Q-Q) plot (b) of GWAS for LN_AFW.** Negative log_10_-transformed *P* values from the compressed mixed linear model were plotted against SNPs position on each of the 12 chromosomes. The horizontal dashed line indicates a genome-wide significance threshold of 3.1×10^-6^.


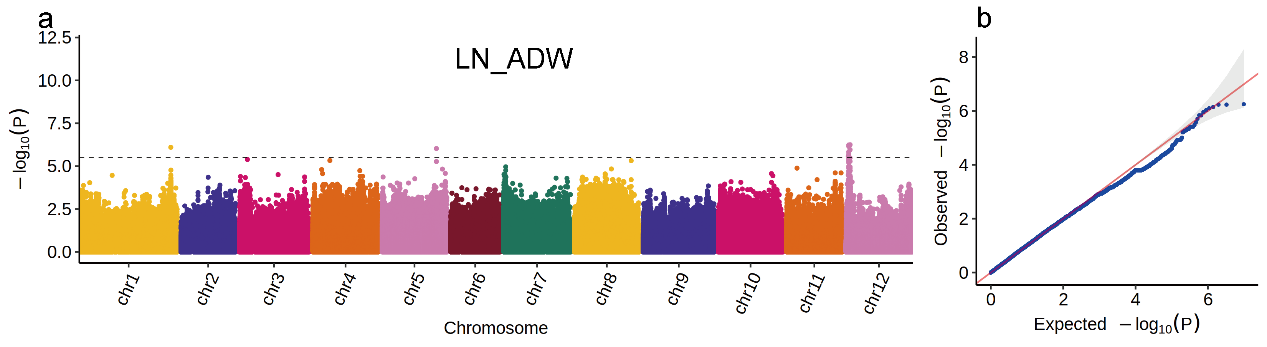


**Supplementary Figure 13: Manhattan plot (a) and Quantile-Quantile (Q-Q) plot (b) of GWAS for LN_ADW.** Negative log_10_-transformed *P* values from the compressed mixed linear model were plotted against SNPs position on each of the 12 chromosomes. The horizontal dashed line indicates a genome-wide significance threshold of 3.1×10^-6^.


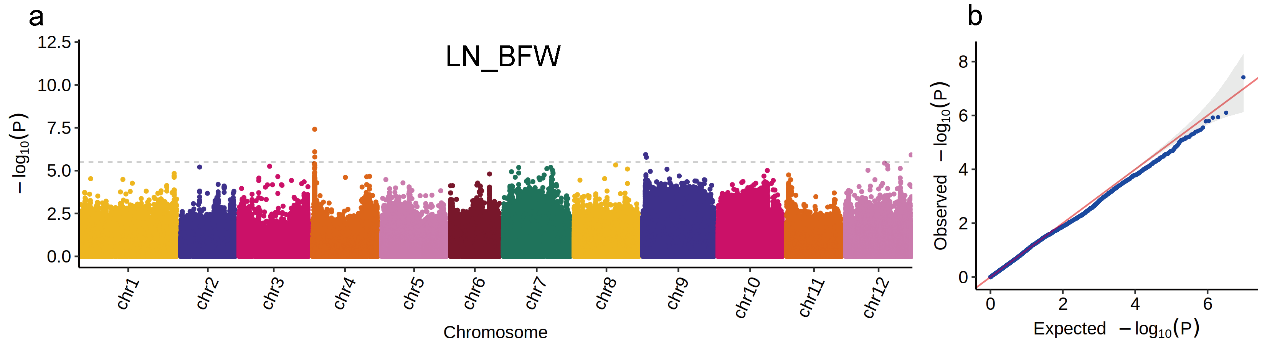


**Supplementary Figure 14: Manhattan plot (a) and Quantile-Quantile (Q-Q) plot (b) of GWAS for LN_BFW.** Negative log_10_-transformed *P* values from the compressed mixed linear model were plotted against SNPs position on each of the 12 chromosomes. The horizontal dashed line indicates a genome-wide significance threshold of 3.1×10^-6^.


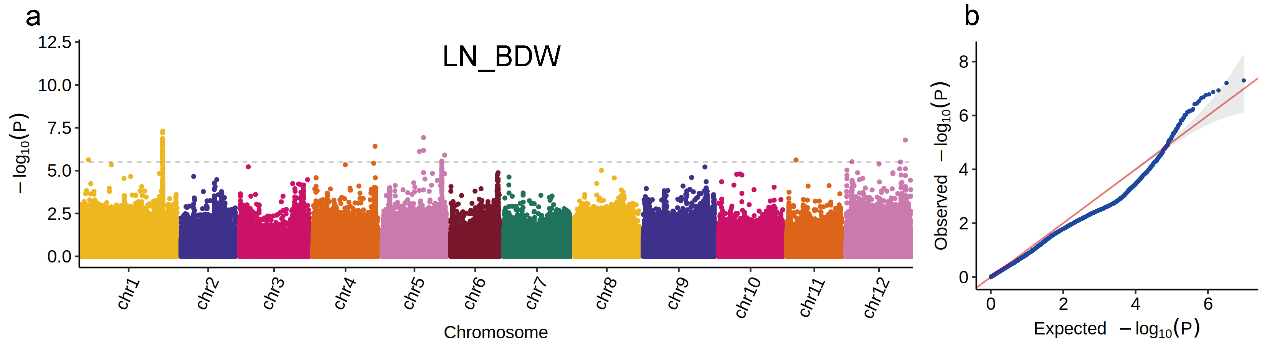


**Supplementary Figure 15: Manhattan plot (a) and Quantile-Quantile (Q-Q) plot (b) of GWAS for LN_BDW.** Negative log_10_-transformed *P* values from the compressed mixed linear model were plotted against SNPs position on each of the 12 chromosomes. The horizontal dashed line indicates a genome-wide significance threshold of 3.1×10^-6^.


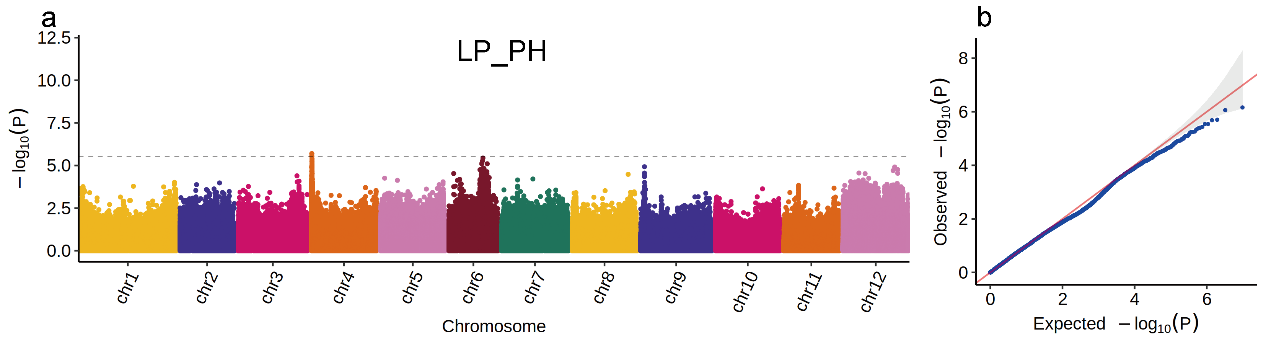


**Supplementary Figure 16: Manhattan plot (a) and Quantile-Quantile (Q-Q) plot (b) of GWAS for LP_PH.** Negative log_10_-transformed *P* values from the compressed mixed linear model were plotted against SNPs position on each of the 12 chromosomes. The horizontal dashed line indicates a genome-wide significance threshold of 3.1×10^-6^.


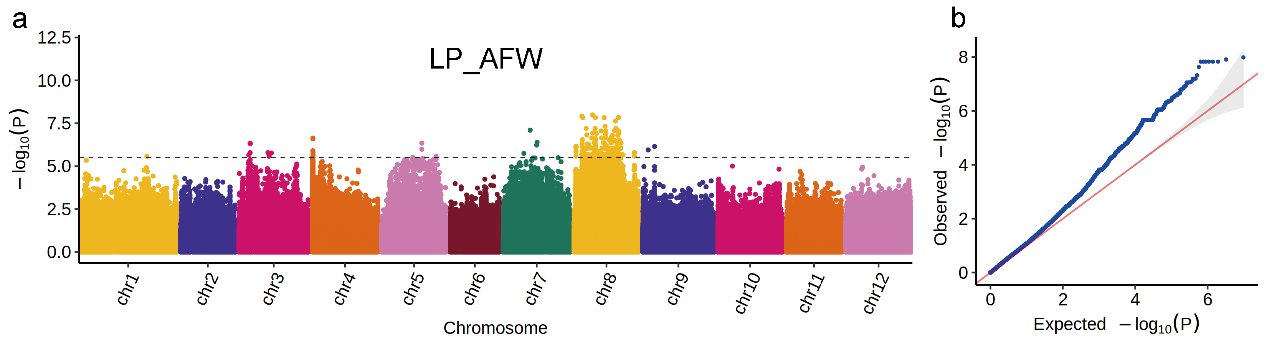


**Supplementary Figure 17: Manhattan plot (a) and Quantile-Quantile (Q-Q) plot (b) of GWAS for LP_AFW.** Negative log_10_-transformed *P* values from the compressed mixed linear model were plotted against SNPs position on each of the 12 chromosomes. The horizontal dashed line indicates a genome-wide significance threshold of 3.1×10^-6^.


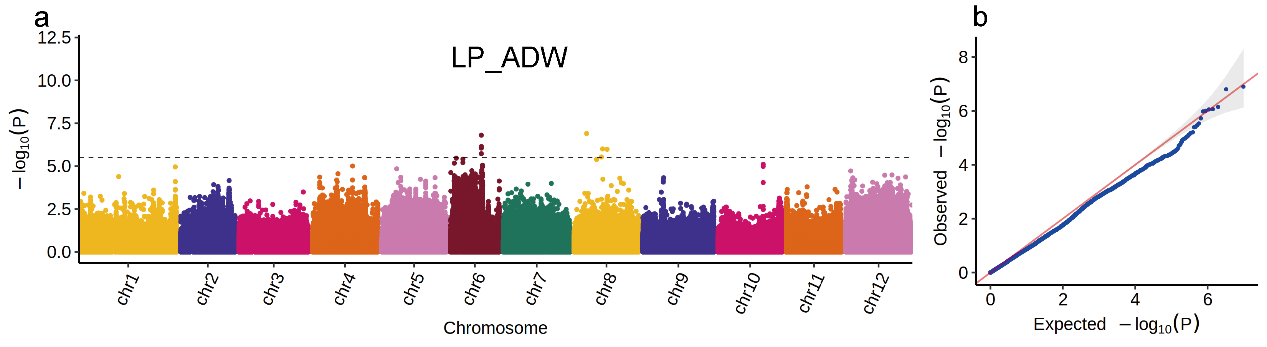


**Supplementary Figure 18: Manhattan plot (a) and Quantile-Quantile (Q-Q) plot (b) of GWAS for LP_ADW.** Negative log_10_-transformed *P* values from the compressed mixed linear model were plotted against SNPs position on each of the 12 chromosomes. The horizontal dashed line indicates a genome-wide significance threshold of 3.1×10^-6^.


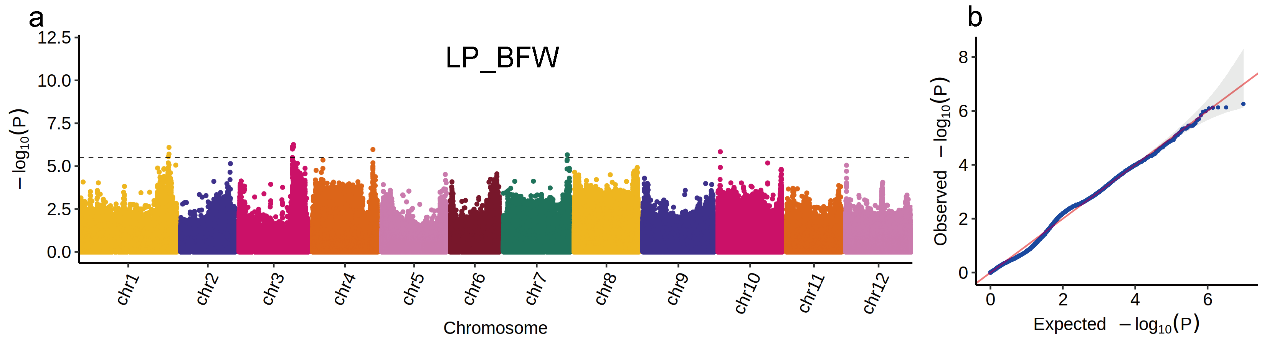


**Supplementary Figure 19: Manhattan plot (a) and Quantile-Quantile (Q-Q) plot (b) of GWAS for LP_BFW.** Negative log_10_-transformed *P* values from the compressed mixed linear model were plotted against SNPs position on each of the 12 chromosomes. The horizontal dashed line indicates a genome-wide significance threshold of 3.1×10^-6^.

**
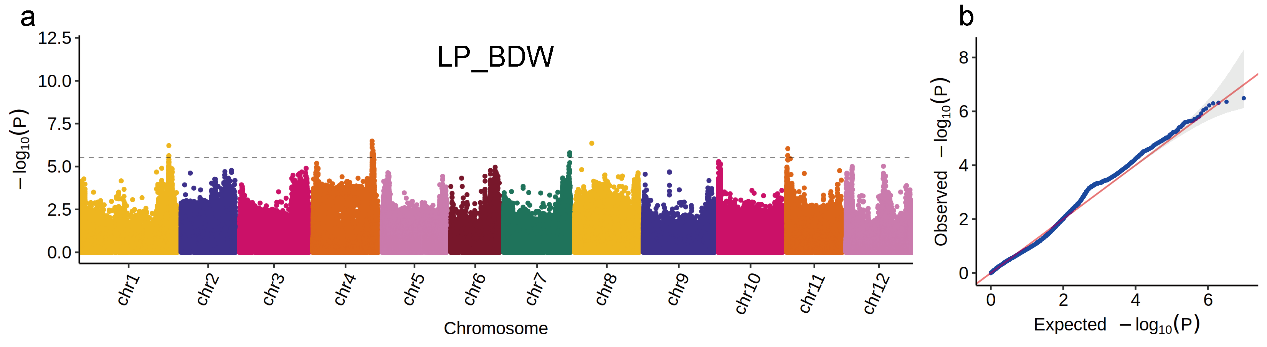
**

**Supplementary Figure 20: Manhattan plot (a) and Quantile-Quantile (Q-Q) plot (b) of GWAS for LP_BDW.** Negative log_10_-transformed *P* values from the compressed mixed linear model were plotted against SNPs position on each of the 12 chromosomes. The horizontal dashed line indicates a genome-wide significance threshold of 3.1×10^-6^.


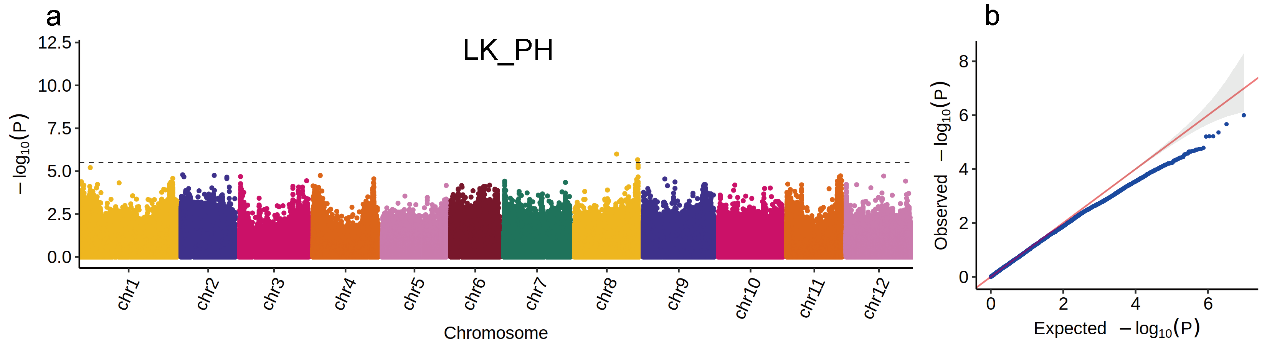


**Supplementary Figure 21: Manhattan plot (a) and Quantile-Quantile (Q-Q) plot (b) of GWAS for LK_PH.** Negative log_10_-transformed *P* values from the compressed mixed linear model were plotted against SNPs position on each of the 12 chromosomes. The horizontal dashed line indicates a genome-wide significance threshold of 3.1×10^-6^.


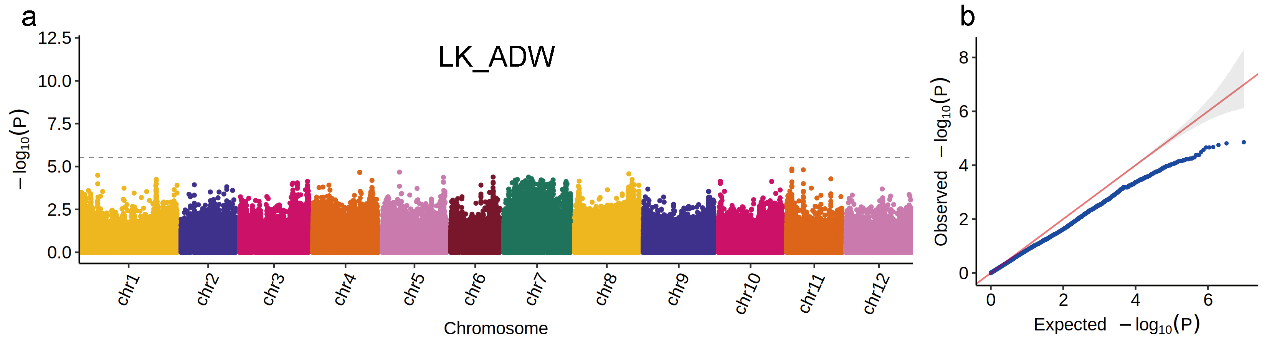


**Supplementary Figure 22: Manhattan plot (a) and Quantile-Quantile (Q-Q) plot (b) of GWAS for LK_ADW.** Negative log_10_-transformed *P* values from the compressed mixed linear model were plotted against SNPs position on each of the 12 chromosomes. The horizontal dashed line indicates a genome-wide significance threshold of 3.1×10^-6^.


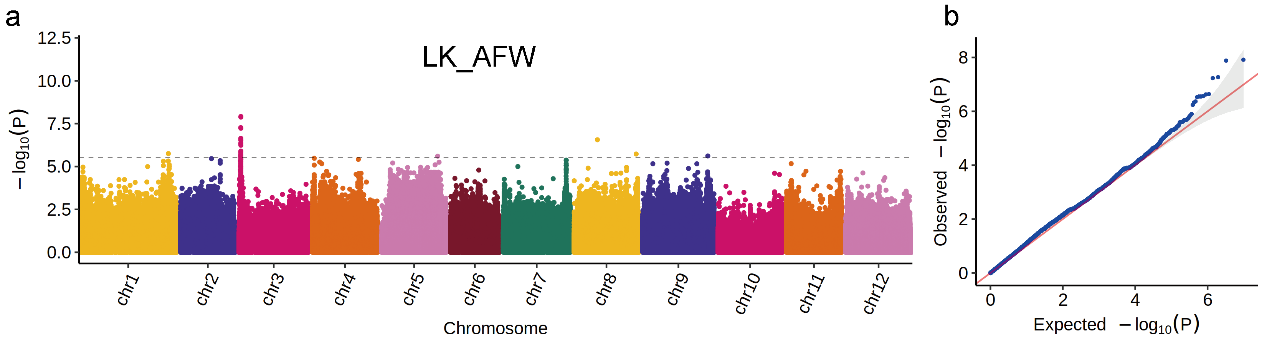


**Supplementary Figure 23: Manhattan plot (a) and Quantile-Quantile (Q-Q) plot (b) of GWAS for LK_AFW.** Negative log_10_-transformed *P* values from the compressed mixed linear model were plotted against SNPs position on each of the 12 chromosomes. The horizontal dashed line indicates a genome-wide significance threshold of 3.1×10^-6^.


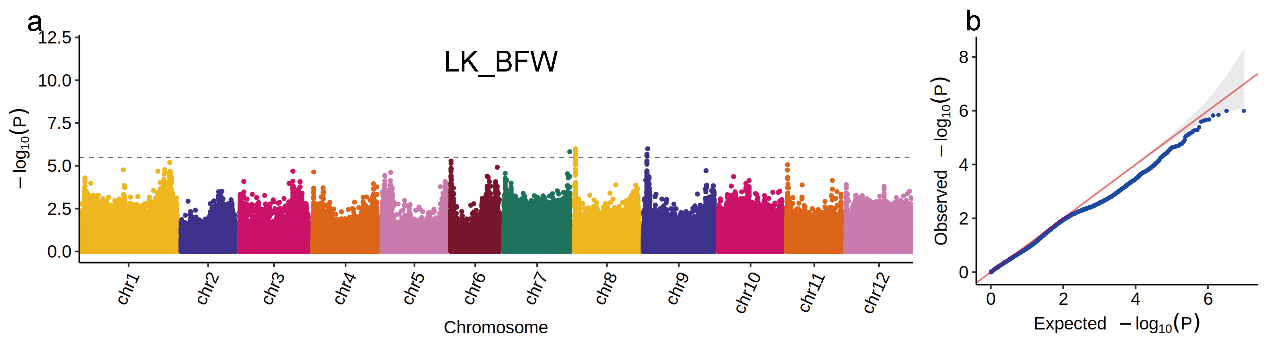


**Supplementary Figure 24: Manhattan plot (a) and Quantile-Quantile (Q-Q) plot (b) of GWAS for LK_BFW.** Negative log_10_-transformed *P* values from the compressed mixed linear model were plotted against SNPs position on each of the 12 chromosomes. The horizontal dashed line indicates a genome-wide significance threshold of 3.1×10^-6^.


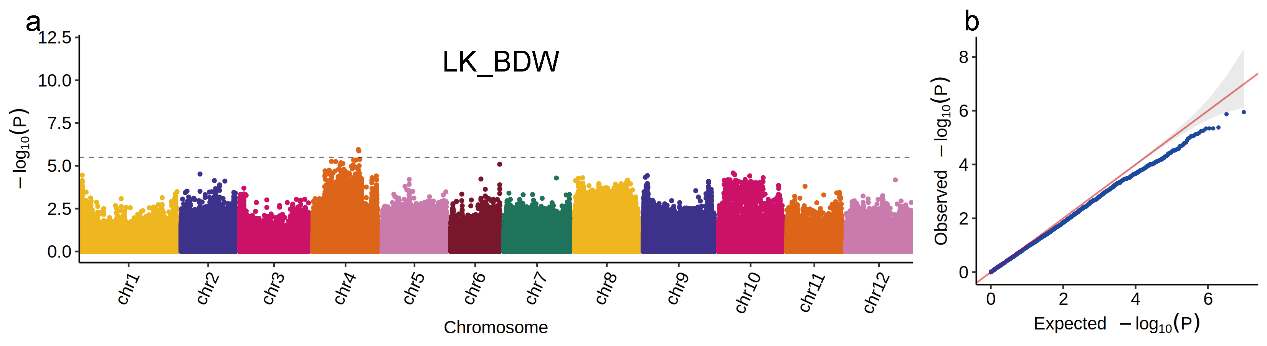


**Supplementary Figure 25: Manhattan plot (a) and Quantile-Quantile (Q-Q) plot (b) of GWAS for LK_BDW.** Negative log_10_-transformed *P* values from the compressed mixed linear model were plotted against SNPs position on each of the 12 chromosomes. The horizontal dashed line indicates a genome-wide significance threshold of 3.1×10^-6^.


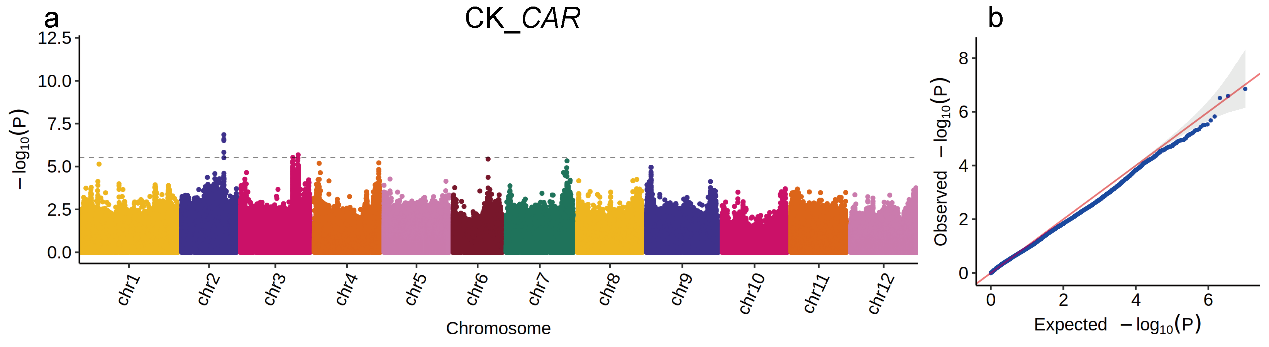


**Supplementary Figure 26: Manhattan plot (a) and Quantile-Quantile (Q-Q) plot (b) of GWAS for CK_*CAR*.** Negative log_10_-transformed *P* values from the compressed mixed linear model were plotted against SNPs position on each of the 12 chromosomes. The horizontal dashed line indicates a genome-wide significance threshold of 3.1×10^-6^.


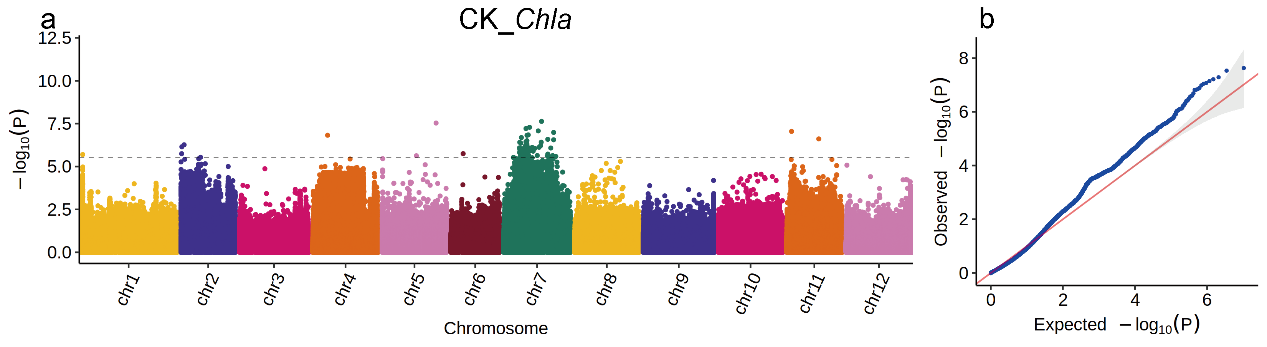


**Supplementary Figure 27: Manhattan plot (a) and Quantile-Quantile (Q-Q) plot (b) of GWAS for CK_*Chla*.** Negative log_10_-transformed *P* values from the compressed mixed linear model were plotted against SNPs position on each of the 12 chromosomes. The horizontal dashed line indicates a genome-wide significance threshold of 3.1×10^-6^.


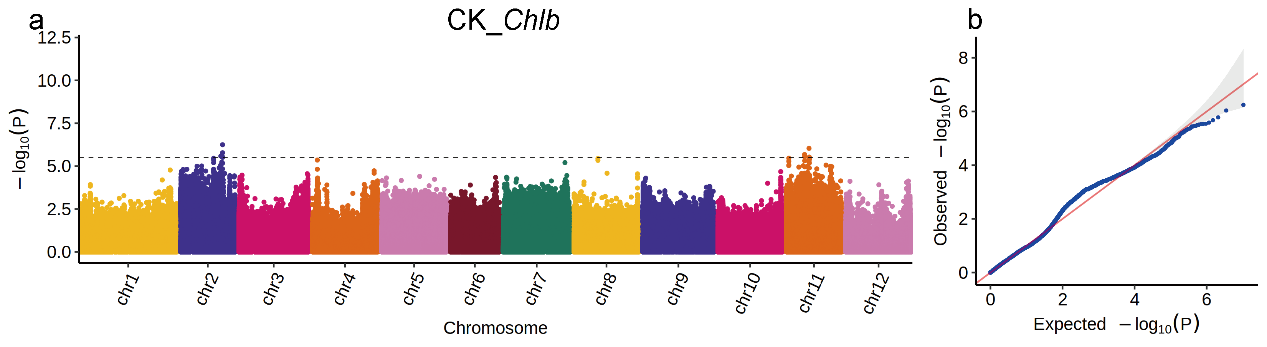


**Supplementary Figure 28: Manhattan plot (a) and Quantile-Quantile (Q-Q) plot (b) of GWAS for CK_*Chlb*.** Negative log_10_-transformed *P* values from the compressed mixed linear model were plotted against SNPs position on each of the 12 chromosomes. The horizontal dashed line indicates a genome-wide significance threshold of 3.1×10^-6^.


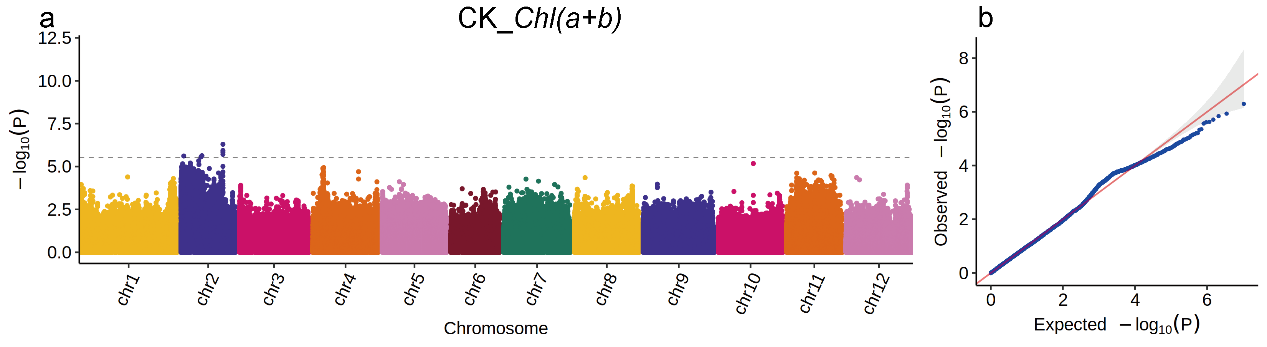


**Supplementary Figure 29: Manhattan plot (a) and Quantile-Quantile (Q-Q) plot (b) of GWAS for CK_*Chl*(*a+b*).** Negative log_10_-transformed *P* values from the compressed mixed linear model were plotted against SNPs position on each of the 12 chromosomes. The horizontal dashed line indicates a genome-wide significance threshold of 3.1×10^-6^.


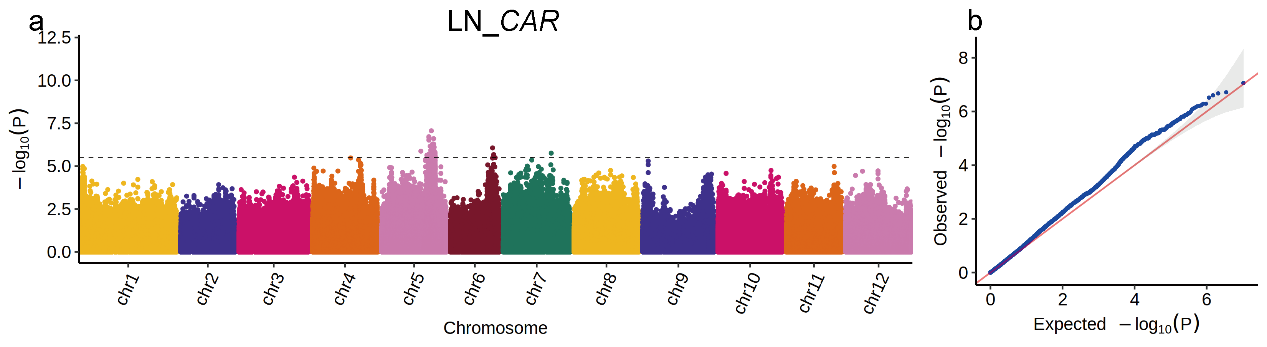


**Supplementary Figure 30: Manhattan plot (a) and Quantile-Quantile (Q-Q) plot (b) of GWAS for LN_*CAR*.** Negative log_10_-transformed *P* values from the compressed mixed linear model were plotted against SNPs position on each of the 12 chromosomes. The horizontal dashed line indicates a genome-wide significance threshold of 3.1×10^-6^.


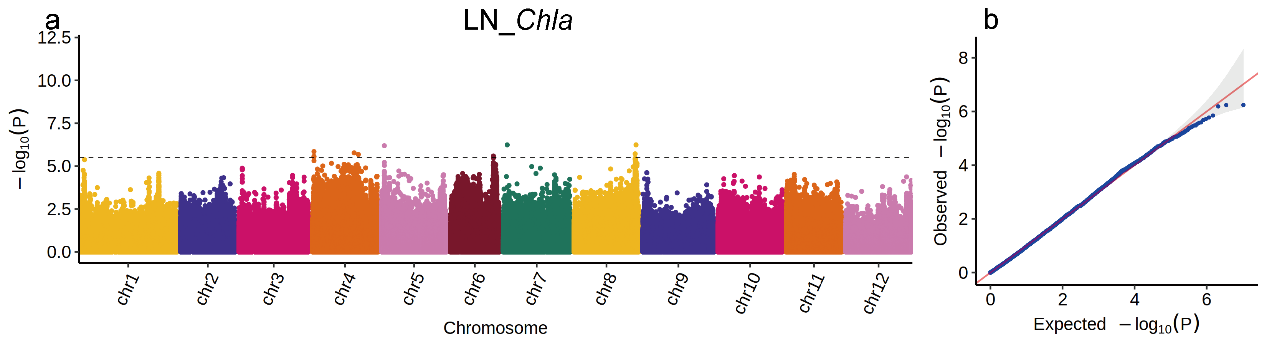


**Supplementary Figure 31: Manhattan plot (a) and Quantile-Quantile (Q-Q) plot (b) of GWAS for LN_*Chla*.** Negative log_10_-transformed *P* values from the compressed mixed linear model were plotted against SNPs position on each of the 12 chromosomes. The horizontal dashed line indicates a genome-wide significance threshold of 3.1×10^-6^.


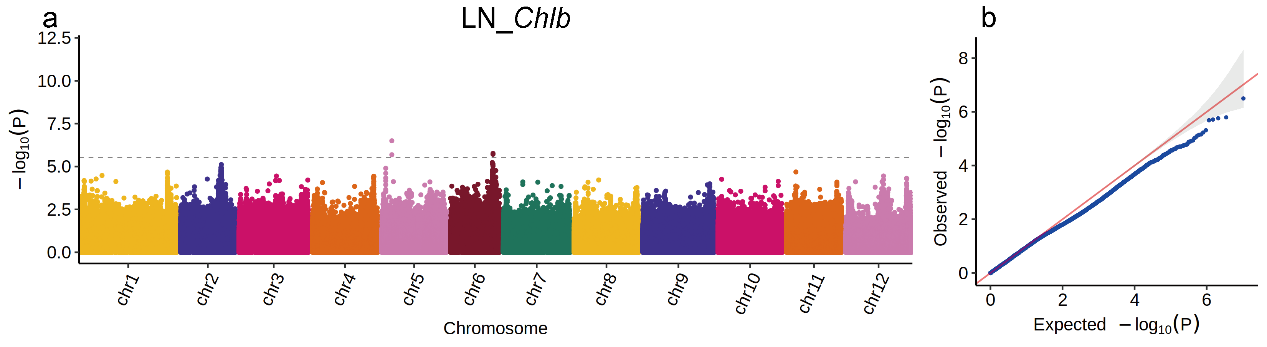


**Supplementary Figure 32: Manhattan plot (a) and Quantile-Quantile (Q-Q) plot (b) of GWAS for LN_*Chlb*.** Negative log_10_-transformed *P* values from the compressed mixed linear model were plotted against SNPs position on each of the 12 chromosomes. The horizontal dashed line indicates a genome-wide significance threshold of 3.1×10^-6^.


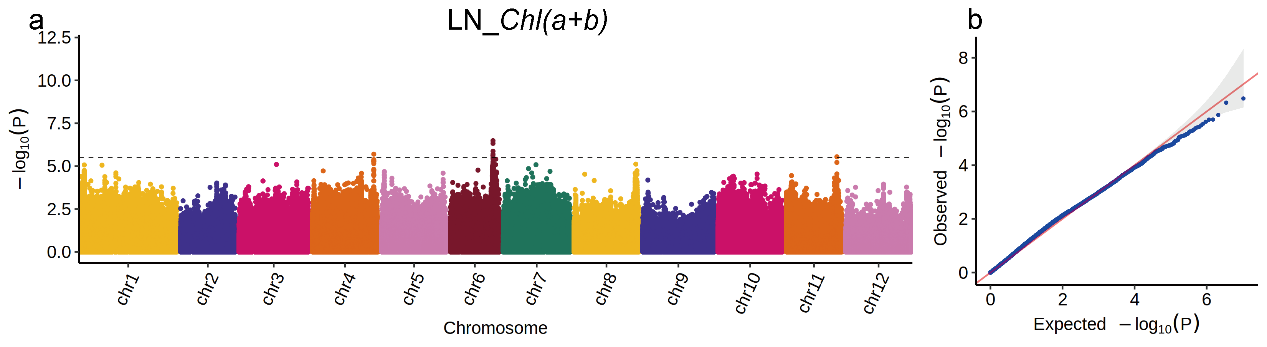


**Supplementary Figure 33: Manhattan plot (a) and Quantile-Quantile (Q-Q) plot (b) of GWAS for LN_*Chl*(*a+b*).** Negative log_10_-transformed *P* values from the compressed mixed linear model were plotted against SNPs position on each of the 12 chromosomes. The horizontal dashed line indicates a genome-wide significance threshold of 3.1×10^-6^.


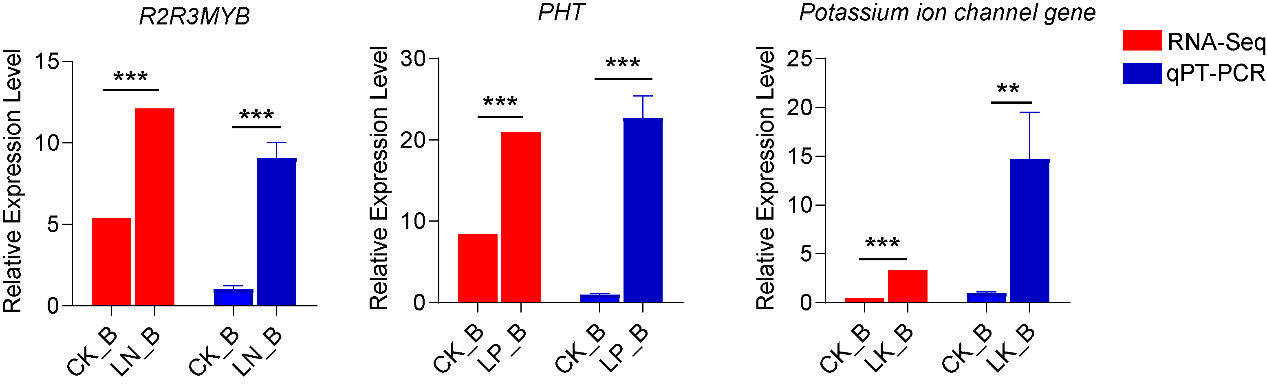


**Supplementary Figure 34: Relative expression levels of marker genes were detected by RNA-Seq and qRT-PCR under different conditions.** *Solyc03g112390* (R2R3MYB transcription factor), *Solyc09g090070* (PHT, Inorganic phosphate transporter), and *Solyc01g010480* (*KCNH8*, potassium voltage-gated channel subfamily H member 8).


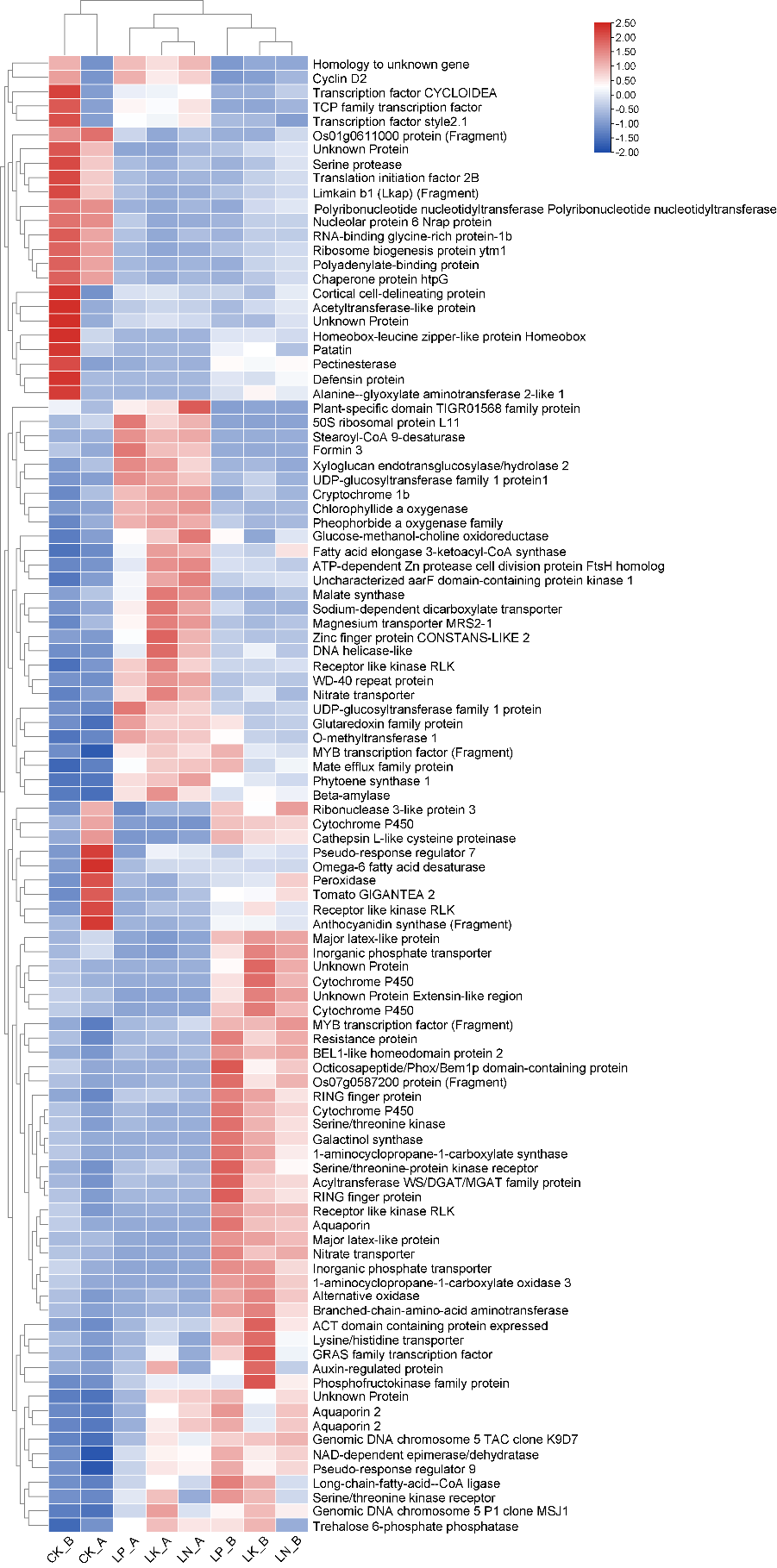


**Supplementary Figure 35: The expression heatmap of 103 common DEGs in above-ground tissues and below-ground tissues under low potassium stress, low nitrogen stress, and low phosphorus stress.**


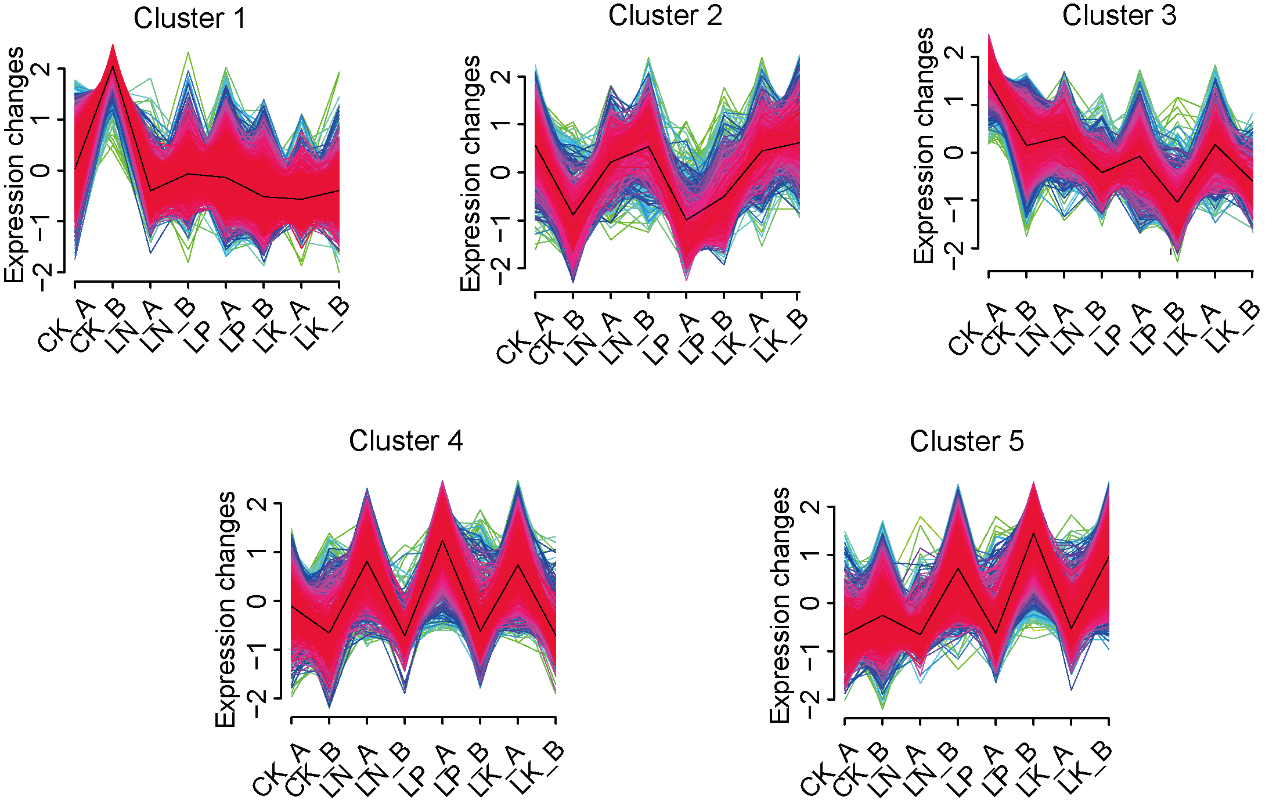


**Supplementary Figure 36: The expression changes of five distinct expression modules in above-ground tissues and below-ground tissues under low potassium stress, low nitrogen stress, and low phosphorus stress.** Cluster 1: Genes that are highly expressed under full nutrient. Cluster 2: Genes that are downregulated under LN, LP, and full nutrient. Cluster 3: Genes that are highly expressed under full nutrient. Cluster 4: Genes that are more highly expressed in the above-ground tissues compared to the below-ground tissues. Cluster 5: Genes showing an expression pattern opposite to that of Cluster 4 under LN, LP and LK stress conditions.

**
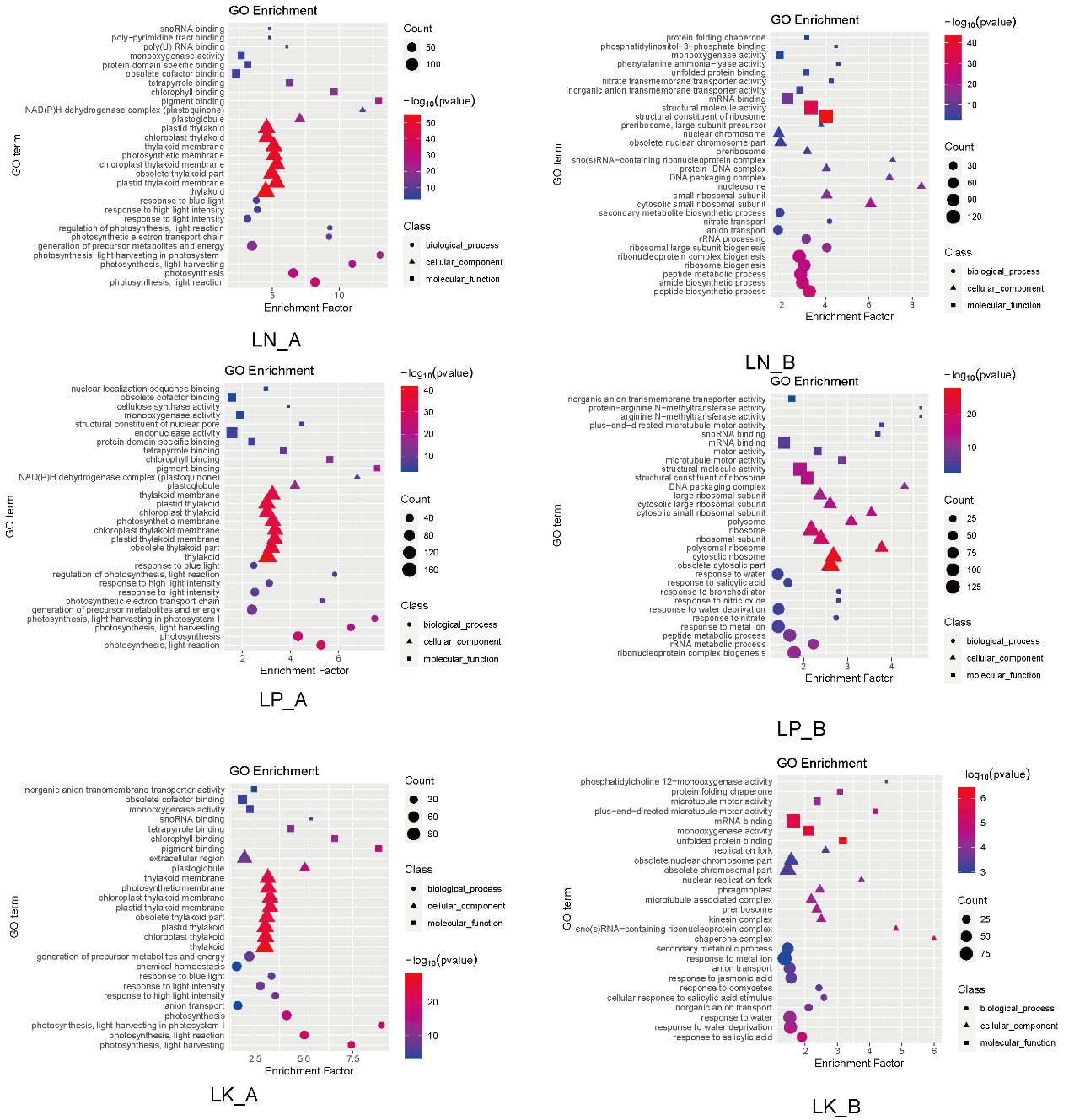
**

**Supplementary Figure 37: The Gene Ontology (GO) enrichment of above-ground tissues and below-ground tissues DEGs under LN, LP, LK treatment.** Using Gfold software with | log2-fold change | > 1 as the threshold, differentially expressed genes (DEGs)


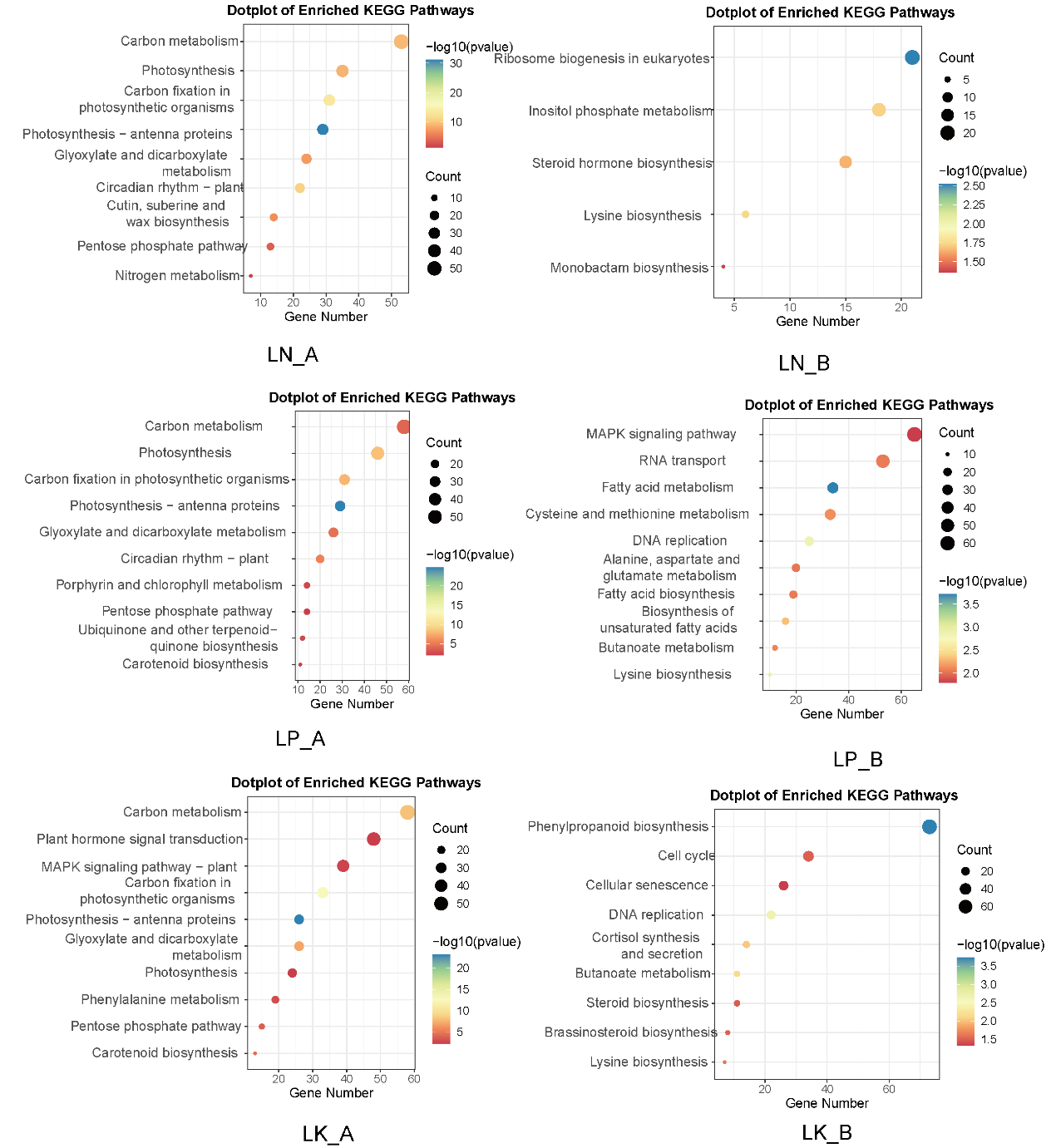


**Supplementary Figure 38: The Kyoto Encvclopedia of Genes and Genomes (KEGG) enrichment of above-ground tissues and below-ground tissues DEGs under LN, LP, LK treatment.** Using Gfold software with |log_2_ fold change| > 1 as the threshold, differentially expressed genes (DEGs)


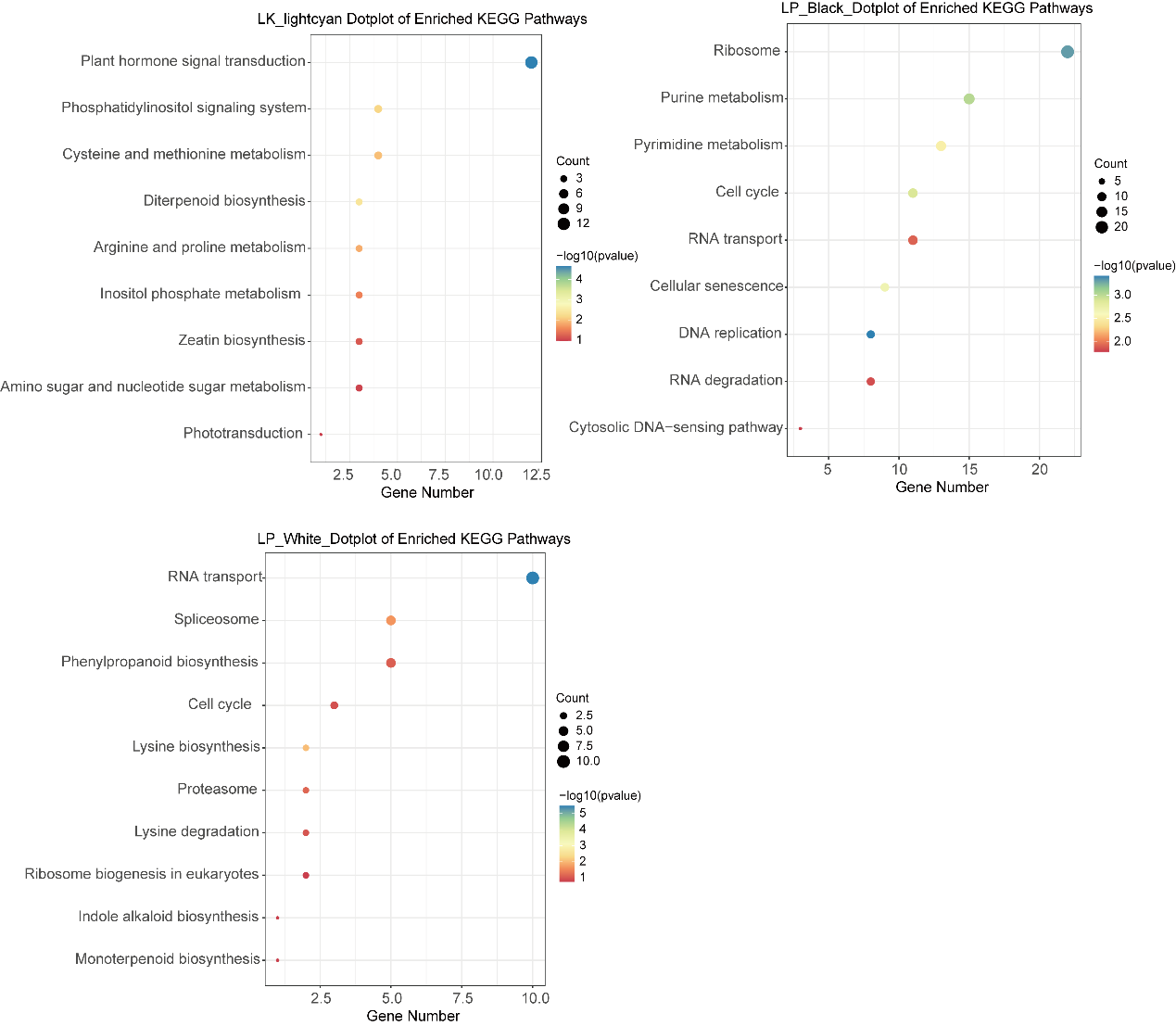


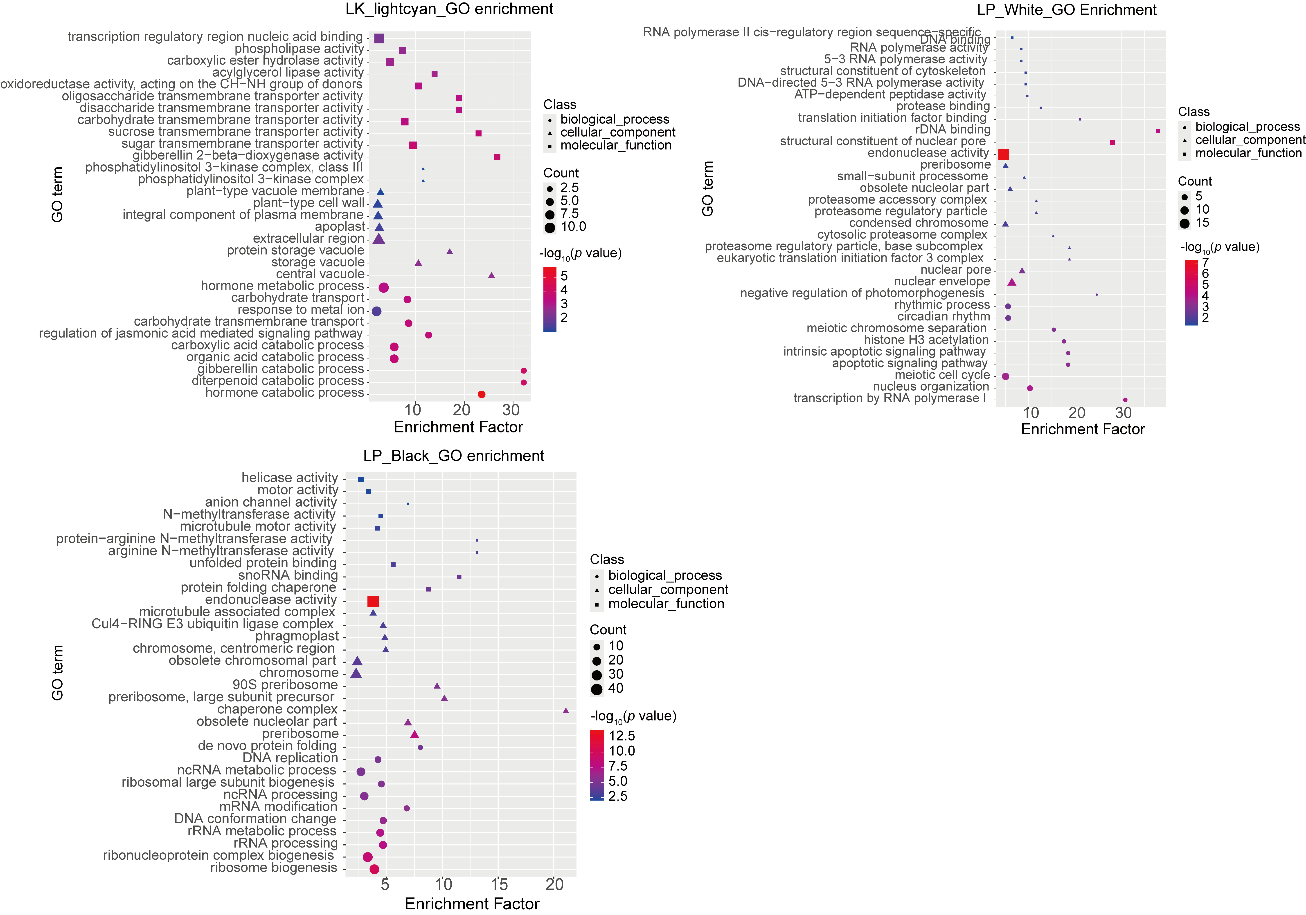


**Supplementary Figure 39:** **KEGG and GO enrichment of WGCNA result.** Above: KEGG enrichment analysis, below: GO enrichment analysis


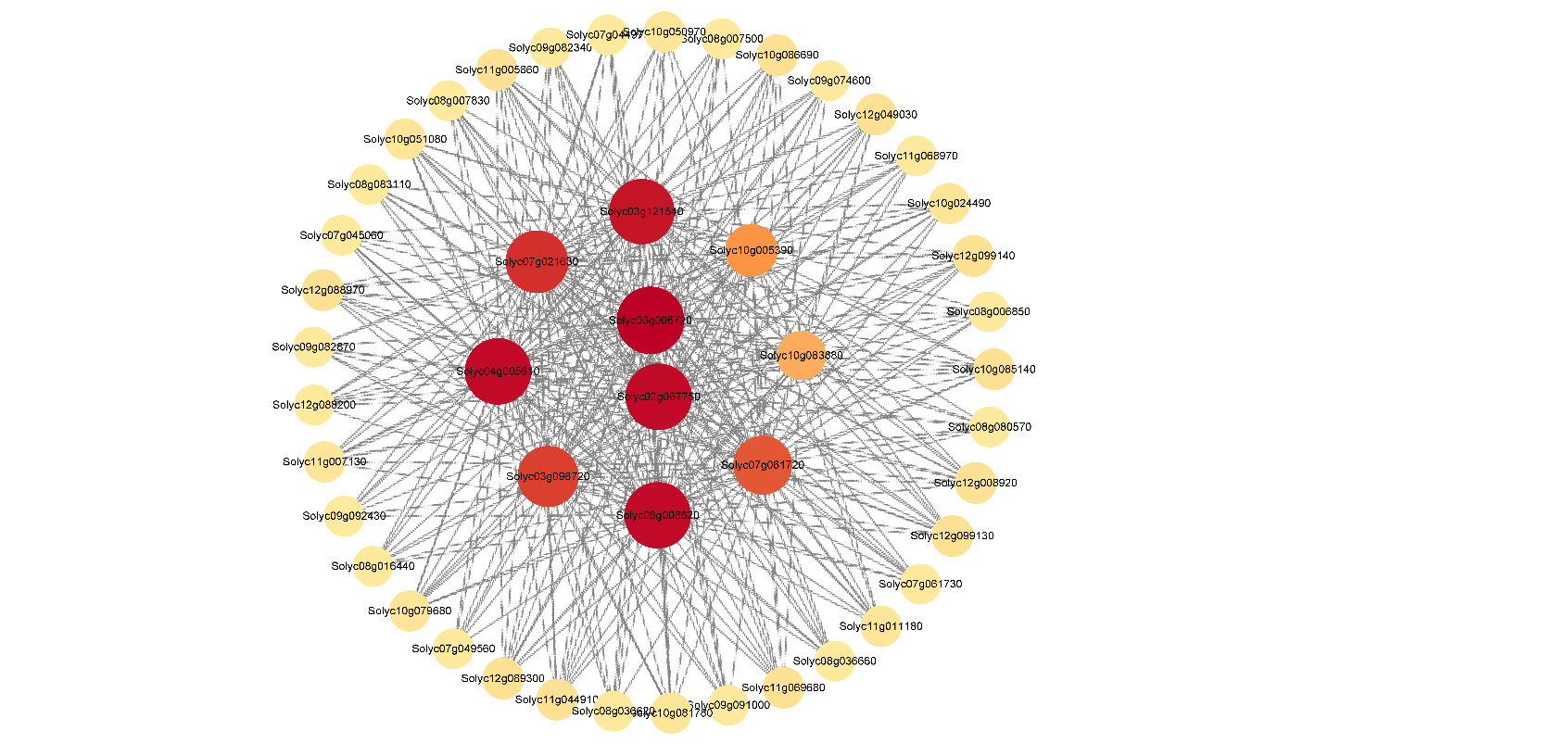


**Supplementary Figure 40: Hub gene co-expression network diagram. Co-expression regulatory network analysis of ‘lightcyan’ module**


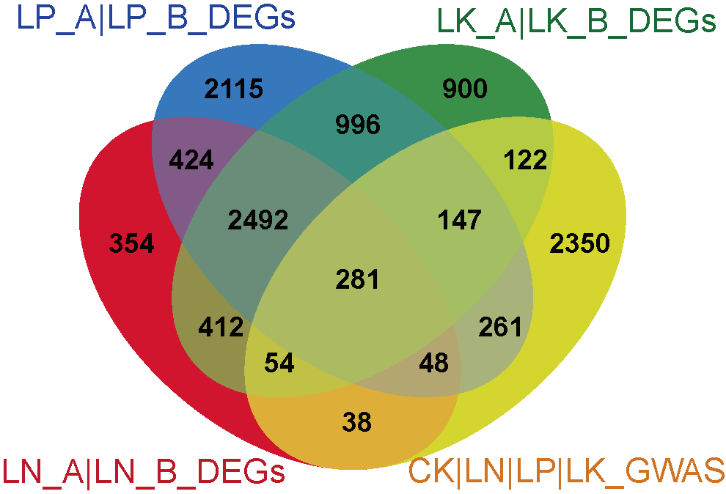


**Supplementary Figure 41: Comparison of genes within the DEGs of above-ground tissues and below-ground tissues in LN, LP, LK and GWAS associated genes.** LN_A|LN_B_DEGs: Compared with CK, DEGs of above-ground tissues and below-ground tissues in LN stress, LP_A|LP_B_DEGs: Compared with CK, DEGs of above-ground tissues and below-ground tissues in LP stress, LK_A|LK_B_DEGs: Compared with CK, DEGs of above-ground tissues and below-ground tissues in LK stress, CK|LN|LP|LK_GWAS: Candidate genes associated with 28 phenotypic datasets were obtained by GWAS.
